# The Consolidated Framework for Implementation Research (CFIR) User Guide: a five-step guide for conducting implementation research using the framework

**DOI:** 10.1186/s13012-025-01450-7

**Published:** 2025-08-16

**Authors:** Caitlin M. Reardon, Laura J. Damschroder, Laura Ellen Ashcraft, Claire Kerins, Rachel L. Bachrach, Andrea L. Nevedal, Ariel M. Domlyn, Jessica Dodge, Matthew Chinman, Shari Rogal

**Affiliations:** 1 VA Center for Healthcare Evaluation, Research, and Promotion, VA Pittsburgh Healthcare System, Pittsburgh, PA USA; 2 Giesel School of Medicine, Dartmouth, Hanover, NH USA; 3 VA Center for Clinical Management Research, VA Ann Arbor Healthcare System, Ann Arbor, MI USA; 4 Implementation Pathways, LLC, Chelsea, MI USA; 5 VA Center for Healthcare Evaluation, Research, and Promotion, Corporal Michael J. Crescenz VA Medical Center, Philadelphia, PA USA; 6 Department of Biostatistics, Epidemiology, and Informatics, Perelman School of Medicine, University of Pennsylvania, Philadelphia, PA USA; 7 Centre for Health Research Methodology, School of Nursing & Midwifery, University of Galway, Galway, Ireland; 8 Health Promotion Research Centre, School of Health Sciences, University of Galway, Galway, Ireland; 9 Department of Psychiatry, University of Michigan Addiction Center, University of Michigan Medical School, Ann Arbor, MI USA; 10 RAND Corporation, Pittsburgh, PA USA; 11 Division of Gastroenterology, Hepatology and Nutrition, Department of Medicine, University of Pittsburgh, Pittsburgh, PA USA; 12 Department of Surgery, University of Pittsburgh, Pittsburgh, PA USA

**Keywords:** Implementation science, Implementation determinants, Implementation outcomes, Barriers, Facilitators, Data collection, Data analysis, User guide

## Abstract

**Background:**

The Consolidated Framework for Implementation Research (CFIR) is a determinant framework that includes constructs from many implementation theories, models, and frameworks; it is used to predict or explain barriers and facilitators to implementation success. CFIR is among the most widely applied implementation science frameworks, and after 15 years of use in the field, the framework was updated based on user feedback obtained via literature review and survey.

Dissemination of the updated CFIR and accompanying outcomes addendum resulted in hundreds of requests from users for further guidance in applying the framework. In addition, observations of potential and actual misuse of CFIR in grant reviews and published manuscripts were the catalyst for the development of this user guide.

As a result, the objective of this article is to provide a user guide and essential tools and templates for using CFIR in implementation research.

**Methods:**

This user guide was generated from the combined wisdom and experience of the CFIR Leadership Team, which includes the lead developers of the original and updated CFIR (LJD, CMR), and has collectively used CFIR in more than 50 projects. The five steps as well as the tools and templates were finalized via consensus discussions.

**Results:**

The five steps below guide users through an entire research project using CFIR and include 1) Study Design; 2) Data Collection; 3) Data Analysis; 4) Data Interpretation; and 5) Knowledge Dissemination. In addition, the article provides a Frequently Asked Questions (FAQs) section based on user queries and six tools and templates: 1) CFIR Construct Example Questions; 2) CFIR Construct Coding Guidelines; 3) Inner Setting Memo Template; 4) CFIR Construct Rating Guidelines; 5) CFIR Construct x Inner Setting Matrix Template; and 6) CFIR Implementation Research Worksheet.

**Conclusion:**

This user guide details how to use CFIR in implementation research, from the design of the study through dissemination of findings, answers frequently asked questions, and offers essential tools and templates. We hope this guidance will facilitate appropriate and consistent application of the framework as well as generate feedback and critique to advance the field.

**Supplementary Information:**

The online version contains supplementary material available at 10.1186/s13012-025-01450-7.

Contributions to the Literature CFIR users often request additional guidance in applying CFIR and misuse of the framework limits the ability to compare findings across projects and advance the field. This article provides

- Guidance for applying CFIR from the design of the study through dissemination of findings;
- Answers to ten frequently asked questions; and
- Essential tools and templates for applying CFIR in implementation research

## Background

The Consolidated Framework for Implementation Research (CFIR) is a determinant framework that includes constructs from many implementation theories, models, and frameworks [1–3]. The overarching aim of CFIR is to predict or explain barriers and facilitators (i.e., the determinants or independent variables) to implementation success (i.e., the outcome or dependent variable). CFIR includes 48 constructs and 19 subconstructs (i.e., determinants) across 5 broad domains: 1) Innovation; 2) Outer Setting; 3) Inner Setting; 4) Individuals: Roles & Characteristics; and 5) Implementation Process [2]. It has been cited over 10,000 times and is among the most widely applied implementation science frameworks [4].

**Fig. 1:**
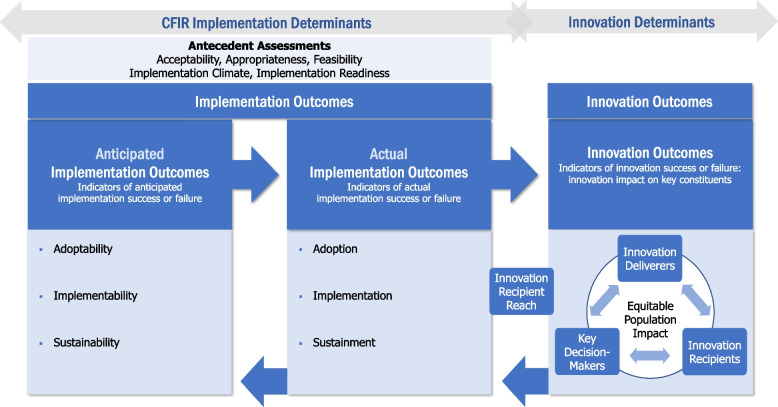
The CFIR Outcomes Addendum*.*This figure has been adapted from the original publication to highlight where Innovation Recipient Reach occurs within the CFIR Outcomes Addendum conceptualization of outcomes

Advances in research rely on users critically reflecting on and refining theories, models, and frameworks [5], and after 15 years of use in the field, CFIR was revised based on user feedback obtained via literature review and survey. The updated CFIR and accompanying CFIR outcomes addendum (Fig. 1) were published in 2022 [2, 6]; updates are described in the Frequently Asked Questions (FAQs) *(see FAQ 1: How did CFIR change in the 2022 update?)*. In addition to changes to the framework, the original CFIR development team expanded into a larger *CFIR Leadership Team (CLT)*, consisting of implementation scientists with a wealth of experience applying CFIR in their own projects as well as training others to use CFIR. CLT members come from a variety of disciplines, including public health, health promotion, evidence-based healthcare, psychology, social work, medicine, and anthropology. The CLT meets twice a month with the goal advancing CFIR scholarship and recently secured a 3-year grant from the Veterans Affairs (VA) Quality Enhancement Research Initiative (QUERI) to create a CFIR “learning hub,” which will provide trainings, consultations, and mentoring for VA researchers and practitioners. Much of this guidance and training will be archived on the CFIR technical assistance website at www.cfirguide.org and will be applicable to a non-VA audience.

Since the CLT began disseminating the updated CFIR and accompanying outcomes addendum, we have received hundreds of requests from researchers, trainees, and seminar attendees for further guidance in applying the framework. In addition, our observations of potential and actual misuse of CFIR in grant reviews and published manuscripts were the catalyst for the development of this user guide. As a result, the objective of this article is to provide a user guide and tools and templates for using CFIR in implementation research to assess determinants before, during, or after implementation of an innovation *(see FAQ 2: What is an “innovation”?)*. Given this scope, it is important to carefully consider if CFIR is right for your project *(see FAQ 3: How do I know if CFIR is right for my project?)* and to understand that CFIR was not designed to guide development of innovations *(see FAQ 4: How do I use CFIR to guide development of an innovation?)* nor to specify the process of implementation *(see FAQ 5: How do I use CFIR to guide the process of implementation?)*. This guide assumes the reader has a basic understanding of foundational implementation terms and concepts [7–12].

## Methods

This user guide was generated from the combined wisdom and experience of the CLT, which includes the lead developers of the original and updated CFIR (LJD, CMR), and has collectively used CFIR in more than 50 projects. While most projects were conducted in US healthcare settings, the team also has experience using CFIR in non-healthcare settings (e.g., public health, education) and outside the US (e.g., Ireland, England), and has led trainings and workshops worldwide. The team began the process with review of the seminal CFIR papers [1, 2, 6] as well as the guidance, tools, and templates previously available on www.cfirguide.org based on the original CFIR.

While the five steps broadly reflect application of determinant frameworks in implementation science [3, 13], the tools and templates are CFIR specific and based on constructs in the updated CFIR.

The five steps as well as the tools and templates were initially drafted by a CFIR developer (CMR) and at least one other team member (see authors’ contributions), reviewed by each remaining team member asynchronously, and finalized via hour long synchronous consensus discussions facilitated by the team project manager. These discussions occurred every other week from January 2024 – January 2025. The manuscript and files each invite additional feedback from users, reflecting the iterative and evolving nature of CFIR as implementation science advances. Note: As is often the case in implementation science literature, we use of the term “implementation” to encompass the full range of outcomes related to adoption, implementation, and sustainment.

## Results

The five steps below guide users through an entire project using CFIR, from the design of the study through dissemination of findings. To ensure successful use of CFIR, we recommend having a qualitative methodologist and/or analyst with experience in implementation science methods and/or using CFIR. Readers can also visit the CFIR technical assistance website at www.cfirguide.org for updates and to provide feedback to the CLT.

### Step 1: Study Design

#### 1A: Define Research Question and Implementation Outcome

Users must first define their research question; CFIR is a determinant framework that can be used prospectively to assess determinants of *anticipated* implementation outcomes (outcomes that have not yet occurred) and/or retrospectively to assess determinants of *actual* implementation outcomes (outcomes that have occurred) [6]. Some projects use CFIR both prospectively and retrospectively, i.e., both looking back to explain current outcomes and looking forward to predict future outcomes. Consequently, users must define their research question to appropriately collect and analyze data using CFIR.

The CFIR outcomes addendum broadly conceptualizes implementation outcomes as measuring the success or failure of implementation, i.e., *the innovation being implemented and delivered as intended in the Inner Setting. Anticipated* implementation outcomes are based on perceptions or measures of the likelihood of future implementation success or failure. These outcomes are prospective; constellations of CFIR determinants across domains may predict these outcomes. *Actual* implementation outcomes are based on perceptions or measures of current (or past) implementation success or failure. These outcomes are retrospective; constellations of CFIR determinants across domains may explain these outcomes [6] (Table 1).

**Table 1:** Prospective and Retrospective Example Research Questions and Implementation Outcomes

Research Question	Temporality	CFIR Implementation Determinant Domains	Implementation Outcome(s)
What barriers and facilitators influence *anticipated* implementation outcomes?	Prospective Assessment (i.e., before adoption, implementation, or sustainment occurs)	Barriers and facilitators related to the Innovation, Outer Setting, Inner Setting, Individuals, and Implementation Process	Adoptability, Implementability, Sustainability
What barriers and facilitators influenced *actual* implementation outcomes?	Retrospective Assessment (i.e., after adoption, implementation, or sustainment has occurred)		Adoption, Implementation, Sustainment

Implementation (and innovation) outcomes (and how they are measured) are project-specific and therefore outside the scope of this guide *(see FAQ 6: What is the most appropriate implementation outcome for my project? and FAQ 7: What is the most appropriate innovation outcome for my project?)*. However, it is critical that each project identify an appropriate implementation outcome. This allows users to identify constructs that distinguish between implementation success or failure – constructs that are “difference-makers” – highlighting the most important barriers to be addressed by future implementation strategies* (see FAQ 8: How do I use CFIR to select implementation strategies?)* or explaining how implementation strategies and constructs interact *(see FAQ 9: How do I use CFIR to compare the effectiveness of different implementation strategies?)*. Finally, implementation is not successful unless it is equitable. Ensuring equitable implementation success requires use of equity-focused implementation process models and measurement frameworks [3, 14–16].

#### 1B: Define CFIR (Implementation Determinant) Domains

CFIR implementation determinants capture barriers and facilitators across five broad domains: 1) Innovation; 2) Outer Setting; 3) Inner Setting; 4) Individuals: Roles & Characteristics; and 5) Implementation Process [2]. Updated guidance urges users to clearly define each domain as well as the boundaries between domains specific to each project. This allows users to make accurate attribution to implementation outcomes [17] and thus identify appropriate areas for future intervention. For example, if the boundary between the innovation and implementation process is not clearly defined and implementation fails, it will be impossible to know if implementation failed due to characteristics of the innovation (i.e., there was something wrong with the innovation) or the implementation strategy(s) (i.e., there was something wrong with the implementation strategy(s)) (Table 2).

**Table 2:** CFIR Implementation Determinant Domains

**Domain**	**Guiding Questions**
**Innovation**	What is the **innovation** being implemented and evaluated? What are its components and features [85, 86]? What is the boundary between the innovation and the process or strategy being used to implement the innovation? What is the (intended) **innovation** **outcome** for: Innovation Recipients Innovation Deliverers High-Level Leaders/Key Decision-Makers
**Individuals: Roles & Characteristics**	Who are the **individuals** involved with implementing, delivering, and/or receiving the innovation? What are their **roles**? What are their **characteristics**?
**Inner Setting & Outer Setting**	**Where** is implementation and delivery of the innovation occurring? What is the boundary between the Inner Setting (the unit of analysis and location where the innovation is being implemented) and the Outer Setting (the area outside of the Inner Setting)?
**Implementation Process**	What is the **implementation process**? Is implementation being guided by a specific implementation strategy or process model [3] (e.g., Knowledge to Action Framework [87], Getting To Outcomes [65], or Getting To Implementation [66])? What are its components and features? What is the boundary between the innovation and the process or strategy being used to implement the innovation?

### Step 2: Data Collection

#### 2A: Determine Data Collection Approach

Both qualitative or quantitative methods can be used to collect data on CFIR determinants and often projects integrate both and use mixed methods. While data collection on CFIR determinants often relies on using qualitative methods, such as semi-structured interviews or focus groups, additional approaches using quantitatively-focused surveys with open-ended text boxes have been developed more recently to complement interview methods [18–20]. There are pros and cons to every data collection approach. For example, CFIR surveys may be less resource intensive for the project team and decrease participant burden, thus potentially allowing for wider participation; however, they will yield little or no qualitative data and instruments have yet to be widely validated. In addition, survey questions rely on a priori questions and assumptions, whereas qualitative methods allow interviewers to ask new questions in direct response to answers. Table 3 includes tradeoffs and information on the three most common data collection approaches used by members of the CLT [21].

**Table 3:** Data Collection & Analysis: Trade-offs based on approach^a^

Approaches	Approach 1	Approach 2	Approach 3
	**Qualitative**		**Quantitative**
Data Collection	Interviews		Surveys
Data Analysis	In-Depth Qualitative Analysis • Coding: CFIR-based deductive and inductive coding of interview transcripts using qualitative software • Data Aggregation: Inner Setting memos containing full data set • Rating: Strength and valence assessments based on construct summaries in Inner Setting memos	Rapid Qualitative Analysis • Coding: CFIR-based deductive and inductive coding of detailed interview note summaries and audio recordings • Data Aggregation: Inner Setting matrix column containing summarized data set • Rating: Strength and valence assessments based on construct summaries in matrix	Quantitative Analysis^b^ • Descriptive and inferential statistics examining associations with inner setting characteristics
**Tradeoffs**	**Qualitative**		**Quantitative**
Participant Burden	High (time to complete interview)	High (time to complete interview)	Low (time to complete survey)
Analyst Hours	High	Medium	Low-Medium
Analyst CFIR Expertise	Medium–High	High (simultaneous data collection and coding with no transcript)	Low
Transcription Delay & Cost	Yes	No	N/A
Level of Detail	High (transcript & recording; lengthy quotations)	Medium–High (recording only; short quotations)	No or limited qualitative data
Rigor	High	High	Medium (survey not validated against interview)

^a^Table adapted from Rapid versus traditional qualitative analysis using the Consolidated Framework for Implementation Research (CFIR) [41, 42] and Applying the Consolidated Framework for Implementation Research in Implementation Science: Theory and Application, edited by Per Nilsen [21]

^b^When surveys include open-ended text boxes, qualitative analysis may also be used: 1) Coding: CFIR-based deductive and inductive coding of open-ended text boxes; 2) Rating: Strength and valence assessments of open-ended text boxes

#### 2B: Develop Data Collection Instruments

*We do not recommend including a question about every CFIR construct in data collection instruments*. In addition to increasing the length of the instrument, which adds burden for participants, not all constructs are relevant for every project. After defining the research question, each construct should be assessed for its likelihood of 1) being a potential barrier or facilitator to the innovation being implemented and delivered or 2) having sufficient variation across the units of analysis (i.e., the Inner Settings). Identifying relevant constructs may be completed by:

- Conducting informal interviews, surveys, or group deliberations with project team members, operational partners, and/or individuals with direct knowledge of the innovation and/or implementing setting
- Reviewing and/or synthesizing the existing literature and implementation theories, models, and frameworks

In addition to CFIR-based questions, open-ended non-construct specific questions must be included to explore the possibility of other determinants or influences not captured in CFIR, e.g., “Why is [Inner Setting] implementing [Innovation]? Examples of open-ended questions for each CFIR construct along with broader implementation questions are available in Additional File 1; these questions must be customized to meet the needs of the project and can then be used in data collection instruments.

Following development of your data collection instrument, we recommend piloting the instrument with project team members, operational partners, and/or individuals with direct knowledge of the innovation and/or implementing setting, and when using qualitative methods, iteratively updating instruments as data collection progresses.

It is important to note that CFIR does not always need to be used to design data collection instruments. Many researchers use open data collection techniques and apply CFIR during data analysis and/or interpretation phases of the project, however, this may increase the risk of missing important determinants [22].

#### 2C: Develop Sampling Strategy

Although CFIR is used to collect data from individuals, information from individual respondents is aggregated to understand constructs at the Inner Setting (i.e., unit of analysis) level. As a result, the first step in developing a sampling strategy is guided by how the Inner Setting is defined for the project. Depending on the objectives of the project, the sample may consist of individuals from a single Inner Setting or dozens of Inner Settings. For example, if you are conducting a quality improvement project to improve implementation in a single Inner Setting, the sample would only include individuals from that location. In contrast, if you are conducting a study to compare determinants across different Inner Settings and/or implementation strategies *(see FAQ 9: How do I use CFIR to compare the effectiveness of different implementation strategies?)*, the sample may include individuals from dozens of locations. The following attributes may be useful to develop a purposeful sample [23] at the Inner Setting level:

- Antecedent Assessments [6, 24], e.g., Organizational Readiness to Change Assessment (ORCA) Scores [25]
- Implementation Outcomes (anticipated or actual) [6]
- Resource allocation, e.g., over- vs. under-resourced settings
- Geographic location, e.g., urban or rural
- Affiliation, e.g., affiliation with an outside agency or university

After selecting the Inner Settings to be assessed, CFIR should be used to collect data from *individuals who have influence and/or power related to implementation and/or delivery of the innovation in the Inner Setting;* purposeful sampling [23] will often include the key decision-makers and individuals implementing and/or delivering the innovation in the Inner Setting, though individuals in external roles, e.g., national level leaders, may sometimes be able to speak to implementation determinants in the Inner Setting [2, 6]. Innovation recipients, e.g., patients or students, are only appropriate to include in the sample of an implementation research study if they have insights into barriers or facilitators to implementation of the innovation in the Inner Setting (*see FAQ 10: Should I use CFIR to collect data from innovation recipients (e.g., patients, students)?)*. Snowball sampling (i.e., asking current respondents for the names of other relevant individuals to collect data from) [23] may help identify all the appropriate individuals. The following attributes may be useful to develop a diverse sample at the individual level:

- Role (in the Inner Setting generally as well with implementation/delivery of the innovation)
- Profession
- Tenure or time in role, i.e., length of time in role or profession
- Demographics

#### 2D: Conduct Data Collection

It is outside the scope of this guide to offer specific direction around collecting data, and there are many high-quality sources on conducting interviews [26, 27] and focus groups [28], completing observations [29–31] and ethnographies [32, 33], obtaining periodic reflections [34], gathering archival data [35], and administering surveys [36].

### Step 3: Data Analysis

#### 3A: Determine Data Analysis Approach

Using CFIR often relies on in-depth qualitative analysis methods: completing deductive (codes derived from CFIR constructs) and inductive (codes derived from the data) coding of transcripts using qualitative software, aggregating coded data by construct in detailed Inner Setting memos (see Additional File 4), and rating each construct as −2 (strong barrier) to + 2 (strong facilitator) to implementation [37–40]. However, newer approaches have evolved including rapid qualitative analysis of interview data [41, 42] and open-ended survey data [43–45] to help reduce time and effort needed for coding and analyses. Many projects use mixed methods, employing both qualitative and quantitative approaches.

The in-depth qualitative CFIR approach is the most resource-intensive, yet yields the most detailed data, and may be best for use in theory building. The rapid qualitative CFIR approach is less resource-intensive, requiring fewer analyst hours and eliminating the cost of transcription, but requires experienced analysts to simultaneously conduct interviews and write and align (“code”) notes with CFIR constructs. This rapid approach yields bigger picture (i.e., less detailed) data compared to more in-depth qualitative analysis [41, 42]. Qualitative data from open-ended text boxes from surveys can be analyzed similar to interview data [43, 44], but likewise typically offers fewer in-depth insights.

It is also possible to analyze CFIR data from surveys quantitatively [18]. Table 3 includes tradeoffs and information on the three most common data analysis approaches used by members of the CLT [21].

#### 3B: Conduct Data Analysis

CFIR provides the initial structure for a qualitative codebook, and detailed coding guidelines for each construct are provided in Additional File 2. These guidelines should be operationalized for each project and further developed throughout the coding process by adding new inductively identified constructs and subconstructs as needed. In addition to coding individual CFIR constructs, analysts can employ causation coding [46] and relationship coding [43, 47] to capture how constructs interact within a project. Causation coding helps identify potential causal links between constructs, while relationship coding captures both unidirectional and bidirectional relationships between constructs.

We recommend having at least two analysts depending on the scope and intensity of the project. Analysts use a consensus-based and iterative process that involves group and independent coding and resolving discrepancies through discussion [48, 49]. If project resources preclude two independent coders for the entire data set, analysts may be able to code independently after achieving consensus on a smaller training dataset (e.g., 10% of transcripts).

After coding, data should be aggregated by unit of analysis, i.e., Inner Setting, and CFIR construct. Queries can be developed in qualitative software to aggregate data and Additional File 3 provides an Inner Setting Memo Template that can help with summarizing data. *Note: If conducting rapid qualitative analysis, data is aggregated during coding in the CFIR Construct x Inner Setting Matrix Template (Additional File 5) *via* a building approach as interviews progress. See previous publication* [41]* and presentation* [42] *for more detail on completing rapid qualitative analysis using CFIR (and how it compares to the in-depth qualitative approach).*

Aggregating data facilitates summarizing and rating data for each construct; ratings are especially useful when there are at least three Inner Settings and there is interest in comparing constructs across Inner Settings based on implementation outcomes. Detailed rating guidelines are provided in Additional File 4. These guidelines should be operationalized for each project to ensure consistency across ratings for each construct and Inner Setting. As with coding, we recommend using a consensus-based approach to finalize ratings. Depending on the project, it may not be helpful to rate the data, or users may wish to collapse ratings into a binary, e.g., barrier vs. facilitator, and only complete the valence (+ vs -) component of rating.

It is outside the scope of this guide to offer specific guidance around analysis of quantitative CFIR data (e.g., from Likert items).

### Step 4: Data Interpretation

#### 4A: Align Implementation Determinants & Outcomes

In order to identify constructs that distinguish between Inner Settings (i.e., unit of analysis) with high and low implementation success – constructs that are “difference-makers” – it is necessary to integrate data on implementation determinants and outcomes. Additional File 5: CFIR Construct x Inner Setting Matrix Template is designed to compare construct ratings (with short summaries of the data and supporting rationale) within and across each Inner Setting in a project. Ratings along with supporting qualitative data can be added for each time point (e.g., pre-implementation, post-implementation) and for each data source (e.g., interviews, surveys, observations), creating a matrix that aggregates the entire data set. This process is similar to a matrixed multiple case study approach [13].

The precise method for consolidating qualitative data and ratings across time points will vary, depending on your research aims. However, aggregate ratings are not a simple average of existing ratings. There is a danger of oversimplifying complex, dynamic descriptions of implementation processes and contexts when applying ratings. We strongly encourage reliance on the underlying qualitative data in addition to the aggregate ratings. We recommend using a consensus-based process, with at least two analysts aggregating ratings and resolving discrepancies through discussion [48, 49]. These discussions provide rich rationale for the ratings; therefore, it is important to clearly document the considerations and final rationale.

#### 4B: Determine Data Interpretation Approach

##### Visual Comparison

With a small sample size, analysts can identify distinguishing constructs visually by sorting the matrix by implementation outcome. For example, in an implementation research study of the VA’s MOVE! Weight Management Program [50], the pattern of ratings (−2, + 1, + 1, + 2, + 2) for *Relative Advantage* appeared to be different between the lower and higher implementation facilities, highlighting that that implementation strategies for MOVE! should include effective communication about the Relative Advantage of the program.

##### Correlational Analysis or Regression Modeling

With sufficient sample size, analysts can identify distinguishing constructs by calculating the correlation between construct ratings and implementation outcomes. For example, in an implementation research study of the VA’s Telephone Lifestyle Coaching (TLC) program [37], distinguishing constructs were identified based on correlational analyses with a priori determined cut-offs. The presence of enthusiastic and capable TLC program *Implementation Leaders* (*r* = 0.65; *p* = 0.03) and effective strategies for *Engaging: Key Stakeholders* (PCPs and other staff) (*r* = 0.66; *p* = 0.03) were strongly correlated with implementation success. In addition, with enough statistical power, analysts can use multivariable regression or other more advanced modeling methods to assess the associations between constructs and outcomes, especially if quantitative measures are used to collect the data. In particular, data reduction methods (e.g., principal component analysis) or tree-based approaches (e.g., XGBoost) may help with wide dataset analysis.

##### Configurational Comparative Methods (CCMs)

With sufficient sample size, analysts can identify “paths” or "recipes” of distinguishing constructs using configurational comparative methods (CCMs), e.g., Coincidence Analysis (CNA), Qualitative Comparative Analysis (QCA) [51, 52]. CCMs consider “equifinality,” meaning that more than one combination of positively (or negatively) rated CFIR constructs may lead to success, as well as causal complexity, where constructs combine in unique ways to produce or not produce an outcome [53]. For example, in an implementation research study on VA access related projects, coincidence analysis found that two CFIR constructs, engagement with *External High-Level Leaders* (i.e., national VA operations) or commitment from *Internal High-Level Leaders* (local facility leadership) were “difference-makers;” the presence of either (not both) of these constructs consistently led to full or partial implementation of an access-related project [54].

### Step 5: Knowledge Dissemination

#### 5A: Determine Knowledge Dissemination Approach

Planning dissemination early can help ensure that you collect data that is meaningful to the audience of interest. Visualization approaches may include a traditional narrative that includes descriptions of the findings and representative quotes, a matrix of key barriers and facilitators with exemplar quotes, a table of frequencies of various barriers and facilitators, a “joint display” in which the visual combination of the qualitative and quantitative results draw out new insights [55], or an implementation research logic model that highlights key barriers and their associations with outcomes and strategies [56]. Case reports are also sometimes used, with one summary for each Inner Setting in the project. Regardless of this decision, we recommend summarizing barriers and facilitators that influence success and any recommendations for next steps or approaches to address barriers and leverage facilitators.

#### 5B: Disseminate Knowledge

It is outside the scope of this guide to offer specific direction around disseminating knowledge, and there are many high-quality sources on responsible [57], effective [58], and innovative [59] knowledge dissemination.

## Discussion

In place of a traditional discussion section, we are including FAQs from users in order to remain in direct conversation with the CFIR Community and answer important questions.

### FAQ 1: How did CFIR change in the 2022 update?

#### CFIR updates include:

- Addition of guidance at the framework-level and domain-level, e.g., to encourage users to customize the framework and define each domain for their project.
- Revisions to domain and construct names and definitions to broaden applicability of CFIR beyond healthcare settings, e.g., replacing the word “patient” with “innovation recipient,” and to correct or clarify information, e.g., adding the word “Innovation” to each construct name in the Innovation Domain.
- Removal and addition of constructs and subconstructs, e.g., removing Implementation Climate and adding the Culture Subconstruct: Deliverer-Centeredness.
- Reorganization of domains and constructs, including relocating constructs, separating single constructs into multiple constructs, and combining multiple constructs into single constructs, e.g., consolidating all relevant roles in the Individuals Domain.

In addition, a companion paper conceptualizing outcomes for use with CFIR was published [6]. These updates were based on user feedback and a full mapping of constructs from the original CFIR to the updated CFIR (as well as the rationale based on user feedback) is available in Additional File 5 in the updated CFIR manuscript [2]. This mapping will be especially useful for teams that started projects using the original CFIR but want to present results using the updated CFIR.

### FAQ 2: What is an “innovation”?

Rogers’ classic Diffusion of Innovation theory defines innovation as an idea, practice, or object that is perceived as new by an individual or other unit of adoption; if an idea seems new within a setting or for an individual, it is an innovation [60]. This is a broad definition and includes any “thing” that is being implemented [8].

While a clearly defined evidence-based innovation (EBI) is foundational in implementation science, and represents the most straightforward use of CFIR, the framework can be adapted to evaluate any “innovation.” For example, CFIR may help identify barriers and facilitators to increasing delivery of a previously implemented innovation, completing a quality improvement project, de-implementing an innovation, or using an implementation strategy. Using CFIR with non-EBI “things” is more challenging and requires additional effort when defining the domains in CFIR.

### FAQ 3: How do I know if CFIR is right for my project?

#### CFIR may be useful when the project meets the following criteria:

1. Your research question includes predicting and/or explaining implementation outcomes based on implementation determinants.
2. The unit of analysis is a defined Inner Setting that will be implementing and delivering the innovation, e.g., hospital, school, city.
3. The team has a methodologist and/or analyst with experience in implementation science methods and/or using CFIR.

### FAQ 4: How do I use CFIR to guide development of an innovation?

CFIR is not designed to guide development of innovations. While many approaches to innovation development include assessing and understanding context [61], we recommend selecting an innovation development framework for your project, e.g., Intervention Mapping [62] or the Framework for Developing and Evaluating Complex Interventions [63]. Following development and implementation of the innovation, CFIR can be used to evaluate determinants to implementation success.

### FAQ 5: How do I use CFIR to guide the process of implementation?

CFIR is not a process model designed to guide the specific steps of implementation [3]; while CFIR includes an Implementation Process Domain, the goal of this domain is to capture the use and quality of these implementation processes as determinants to implementation success, not to directly guide implementation. While CFIR can guide assessment of potential barriers and facilitators to implementation, we recommend selecting a process model to guide implementation, e.g., Quality Implementation Framework [64], Getting To Outcomes [65], or Getting To Implementation [66]. Following implementation of the innovation, CFIR can be used to evaluate determinants to implementation success.

### FAQ 6: What is the most appropriate implementation outcome for my project?

Ideally, your chosen implementation outcome is one that is most proximal to the implementation strategy(s) being used and is an indicator of implementation (i.e., delivery of the innovation) within the Inner Setting.

Both qualitative (e.g., fidelity observations, interviews) and quantitative (e.g., surveys, administrative data) can be used to assess implementation outcomes. Questions that may be useful for assessing anticipated and/or actual implementation outcomes are included in Additional File 1; these questions must be customized to fit each project but can then be used as a part of data collection instruments. We recommend collecting an “objective” measure of implementation that is assessed by an outside evaluator, e.g., fidelity ratings or administrative data, that reflect the extent to which implementation is complete and equitable within and across Inner Settings.

Additional guidance and a mapping of implementation outcomes across RE-AIM [67] and the Implementation Outcomes Framework [68] is available in the CFIR outcomes addendum [6].

### FAQ 7: What is the most appropriate innovation outcome for my project?

Innovation outcomes include the impact of the innovation on recipients, deliverers, and key-decision makers [6] and are innovation specific. While hybrid implementation-effectiveness designs are used to assess both implementation and innovation outcomes [10], measuring innovation effectiveness requires collecting additional data on innovation determinants (e.g., patient-level determinants) and outcomes (e.g., patient-level outcomes). See the CFIR outcomes addendum for more information [6].

### FAQ 8: How do I use CFIR to select implementation strategies?

After identifying potential (or actual) barriers and facilitators to implementation using CFIR, strategies to mitigate barriers and leverage facilitators can be developed and/or identified via several participatory methods such as user-centered design [69], Implementation Mapping [70], or concept mapping [71]. In addition, a tool to help users “match” strategies to barriers was developed using the original CFIR [72], with strategies being drawn from Expert Recommendations for Implementing Change (ERIC) [73, 74].

### FAQ 9: How do I use CFIR to compare the effectiveness of different implementation strategies?

Overall, you will use CFIR as described in this guide. You may find it useful to map components of the implementation strategy to constructs in the Implementation Process Domain, where some of the more common strategies are included as constructs. This will facilitate comparing how components of the implementation strategy appear in the data in each trial arm, i.e., how they manifest and/or interact differently with other constructs based on the implementation strategy used [40].

### FAQ 10: Should I use CFIR to collect data from innovation recipients (e.g., patients or students)?

When collecting data, researchers must be clear about the goal of data collection: 1) to predict and/or explain implementation outcomes based on implementation determinants (this is within the scope of CFIR); or 2) to predict and/or explain innovation outcomes based on innovation determinants (this is outside the scope of CFIR).

CFIR implementation determinants capture Inner Setting-level barriers and facilitators that predict and/or explain *implementation outcomes, i.e., the innovation being implemented and delivered as intended in the Inner Setting.* These determinants are denoted by the gray arrow in Fig. 1 labeled *CFIR Implementation Determinants.* Data (qualitative and/or quantitative) on these determinants is best collected from individuals who have influence and/or power related to implementation (usually folks within the implementing setting); these typically include the key decision-makers and individuals implementing and/or delivering the innovation.

As a result, CFIR is not the appropriate framework to use when collecting data from recipients, unless recipients are also helping to implement and/or deliver the innovation in the Inner Setting. As reflected by Orlando et al., it is disappointing to note that “… while patients are part of the health-care organization and are essential to assessing *intervention [innovation]* effectiveness, they are a less influential component of *implementation* success in health-care settings than administrators and physicians” (emphases added) [75]. Although hospital systems are increasingly prioritizing patient-centered care, convening patient advisory boards, and involving patients in co-design of initiatives [76, 77], these efforts have not yet resulted in true power-sharing between innovation recipients and key decision-makers in the Inner Setting [78].

As a result, direct data collection from recipients does not usually inform implementation outcomes. Instead, data collection from key decision-makers and individuals implementing and/or delivering the innovation about *their* perceptions of recipients (e.g., recipient needs and characteristics), and how those perceptions encourage (or discourage) completing implementation, informs Implementation Outcomes. *Although CFIR is often not appropriate for use with recipients (because they rarely hold roles as key decision-makers or innovation implementers/deliverers in the Inner Setting), we hope that will change. Recipients should have greater influence, authority, and power in systems; the updated CFIR highlighted the importance of implementation teams including innovation recipients (and innovation deliverers) as members. When recipients serve in that role, we strongly encourage using CFIR to collect data about implementation determinants from them – because they are also implementation team members. Ultimately, equitable population impact is only possible when recipients are integrally involved in implementation and all key constituencies share power and make decisions together*.

In contrast to implementation determinants, innovation determinants capture recipient-level characteristics and/or experiences with the innovation that predict and/or explain *innovation outcomes.* These determinants are denoted by the gray arrow in Fig. 1 labeled *Innovation Determinants.* Data (qualitative and/or quantitative) on these determinants is best collected from recipients. Innovation determinants include constructs or measures that are based on the theoretical framework underlying the innovation. For example, in a “small change” weight loss intervention designed for patients, innovation determinants included patient-level demographics, motivation and intention, and self-efficacy because the intervention was guided by social-psychological and goal-conflict theories [79]. This innovation was tested within a randomized clinical trial [80] and a subset of patient characteristics (innovation determinants) were explored in secondary analyses to help explain innovation outcomes [81–84]. *CFIR was not designed to capture these theory-derived determinants of innovation outcomes, and adapting CFIR constructs for this purpose separates them from the underlying organizational theory*.

## Conclusions

This user guide details how to use CFIR in implementation research, from the design of the study through dissemination of findings, and Additional File 6 provides an accompanying worksheet to guide users through each step. In addition, the user guide provides answers to frequently asked questions and offers essential tools and templates, including: CFIR Construct Example Questions, CFIR Construct Coding Guidelines, an Inner Setting Memo Template, CFIR Construct Rating Guidelines, and a CFIR Construct x Inner Setting Matrix Template. We hope this guidance will facilitate appropriate and consistent application of the framework as well as generate feedback and critique to advance the field.

## Supplementary Information

Additional file 1: CFIR Construct Example Questions.

Additional file 2: CFIR Construct Coding Guidelines.

Additional file 3: Inner Setting Memo Template.

Additional file 4: CFIR Construct Rating Guidelines.

Additional file 5: CFIR Construct x Inner Setting Matrix Template.

Additional file 6: CFIR Implementation Research Worksheet.

## Data Availability

Data sharing is not applicable to this article as no datasets were generated or analyzed during the current study.

## Abbreviations

CCMs: Configurational Comparative Methods
CFIR: Consolidated Framework for Implementation Research
CLT: CFIR Leadership Team
CNA: Coincidence Analysis
EBI: Evidence-Based Innovation
ERIC: Expert Recommendations for Implementing Change
FAQs: Frequently Asked Questions
ORCA: Organizational Readiness to Change Assessment
QCA: Qualitative Comparative Analysis
QUERI: Quality Enhancement Research Initiative
VA: Veterans Affairs
