# Supplementary material for: The Consolidated Framework for Implementation Research (CFIR) User Guide: a five-step guide for conducting implementation research using the framework
Source: Implement Sci. 2025 Aug 16;20:39. doi: 10.1186/s13012-025-01450-7 (PMC12357348; doi:10.1186/s13012-025-01450-7)
Supplement: Supplementary file 1 — Additional file 1: CFIR Construct Example Questions. [file 13012_2025_1450_MOESM1_ESM.docx]

# CFIR Construct Example Questions

## Introduction

This document provides **examples** of open-ended questions for each CFIR construct along with broader non-CFIR specific questions. We have provided two types of example questions:

1) Questions for use in prospective studies where data collection occurs pre-implementation to predict implementation outcomes;

2) Questions for use in retrospective studies where data collection occurs post-implementation to understand implementation experiences and explain implementation outcomes.

Questions can also be used during implementation, which may draw from either source depending on the purpose of data collection. When reporting and analyzing results, users should clearly specify the temporality of their project.

We do not recommend including a question for every CFIR construct in data collection instruments; please refer to the CFIR User Guide manuscript for more information on selecting constructs and piloting your instrument.

**These questions are only examples to help users understand and assess the underlying construct**. It is important to customize questions based on your project (i.e., Innovation, Inner Setting) and interviewee (i.e., appropriate, understandable language) as well as time period (i.e., where we use the words implementation and/or delivery in most questions, users will want to replace those words with adoption or sustainment if they are conducting an evaluation before or after implementation). **Please wordsmith and make the questions your own!**

Please remember that CFIR captures determinants to Implementation Outcomes*, i.e.,* ***the innovation being delivered as intended in the Inner Setting****.* Data (qualitative and/or quantitative) on these determinants is best collected from individuals who have influence and/or power related to implementation (usually folks within the implementing setting); these typically include the key decision-makers and individuals implementing and/or delivering the innovation.

See full note at the end of the document regarding using CFIR to collect data from Innovation Recipients (e.g., patients, students).

***Note:*** *This document is the first iteration of updated CFIR construct example questions; please provide feedback regarding this tool via www.cfirguide.org in order for us to improve it!*

## Non-CFIR Specific Questions

| **NON-CFIR SPECIFIC QUESTION** | ***Open-ended non-construct specific questions:*** Questions to explore the possibility of other determinants or influences not captured in CFIR. | | |
| --- | --- | --- | --- |
| **Name** | **Definition** | **Prospective Questions:** These questions will help assess determinants of Anticipated Implementation Outcomes [1]. | **Retrospective Questions:** These questions will help assess determinants of Actual Implementation Outcomes [1]. |
| **Implementation Rationale** | The reason the Inner Setting implemented the innovation. | Why is [INNER SETTING] implementing [INNOVATION]? | Why did [INNER SETTING] implement [INNOVATION]? |
| **Barriers & Facilitators** | General barriers and facilitators to implementation. | What may make implementation more difficult, i.e., what barriers might you experience?  What may make implementation easier, i.e., what facilitators might you experience? | What made implementation more difficult, i.e., what barriers did you experience?  What made implementation easier, i.e., what facilitators did you experience? |
| **Probes** | Questions that can be added to any construct below to obtain additional detail or information. | What influence (if any) might that have on implementation?  What do others in [INNER SETTING] think?  Could you provide an example? | What influence (if any) did that have on implementation?  What did others in [INNER SETTING] think?  Could you provide an example? |

## Innovation Domain Questions

| **I. INNOVATION DOMAIN** | ***Innovation:*** The “thing” being implemented [2], e.g., a new clinical treatment, educational program, or city service.  ***Project Innovation:*** [Document the innovation being implemented, e.g., innovation type, innovation core vs. adaptable components, using a published reporting guideline [3], [4], [5], [6]. Distinguish the innovation (the “thing” that continues when implementation is complete) [2], [7] from the implementation process and strategies used to implement the innovation [8], [9] (activities that end after implementation is complete) [10].]  ***Note:*** *Many constructs in the Innovation Domain may be more relevant pre-adoption of the innovation (when key decision-makers are deciding whether to put the innovation in place or not)* [1] *versus implementation or sustainment of the innovation.* | | |
| --- | --- | --- | --- |
| **CFIR Construct Name** | **Construct Definition** *The degree to which:* | **Prospective Questions:** These questions will help assess determinants of Anticipated Implementation Outcomes [1]. | **Retrospective Questions:** These questions will help assess determinants of Actual Implementation Outcomes [1].  ***Note:*** *Retrospective questions in this domain are written in the past tense to ensure teams capture implementation determinants* ***not*** *implementation outcomes.* |
| A. Innovation Source | The group that developed and/or visibly sponsored use of the innovation is reputable, credible, and/or trustable. | Who developed or is sponsoring [INNOVATION]? To what extent is the [INNOVATION SOURCE] credible? | Thinking back to before or during implementation:  Did you know who developed or was sponsoring [INNOVATION]? To what extent was the [INNOVATION SOURCE] credible? |
| B. Innovation Evidence-Base | The innovation has robust evidence supporting its effectiveness. | What evidence is there that [INNOVATION] will be effective in [INNER SETTING]?   - How robust is the evidence? - What kind of evidence is missing? | Thinking back to before or during implementation:  What evidence was there that [INNOVATION] would be effective in [INNER SETTING]?   - How robust was the evidence? - What kind of evidence was missing? |
| C. Innovation Relative Advantage | The innovation is better than other available innovations or current practice. | How does [INNOVATION] compare to current practice? To [Alternative Innovation]?   - What are advantages and disadvantages of [INNOVATION] compared to current practice? To [ALTERNATIVE INNOVATION]? | Thinking back to before or during implementation:  How did you think [INNOVATION] compared to current practice? To [Alternative Innovation]?   - What did you think were advantages and disadvantages of [INNOVATION] compared to current practice? To [ALTERNATIVE INNOVATION]? |
| D. Innovation Adaptability | The innovation can be modified, tailored, or refined to fit local context or needs. | ***Note:*** *This construct captures the inherent adaptability of the innovation,* ***not*** *the need to adapt nor the process of adapting the innovation.*  To what extent is [INNOVATION] adaptable? | ***Note:*** *This construct captures the inherent adaptability of the innovation,* ***not*** *the need to adapt nor the process of adapting the innovation.*  Thinking back to before or during implementation:  To what extent did you think [INNOVATION] was adaptable? |
| E. Innovation Trialability | The innovation can be tested or piloted on a small scale and undone. | ***Note:*** *This construct captures the inherent trialability and/or reversibility of the innovation,* ***not*** *the need to trial nor the process of trialing the innovation.*  To what extent is [INNOVATION] trialable, i.e., to what extent can it be piloted and undone? | ***Note:*** *This construct captures the inherent trialability and/or reversibility of the innovation,* ***not*** *the need to trial nor the process of trialing the innovation.*  Thinking back to before or during implementation:  To what extent did you think [INNOVATION] was trialable, i.e., to what extent could it be piloted and undone? |
| F. Innovation Complexity | The innovation is complicated, which may be reflected by its scope and/or the nature and number of connections and steps. | ***Note:*** *If respondents start to discuss the complexity of implementation (versus the complexity of the innovation), probe to identify the root causes of implementation complexity, in order to later code the appropriate CFIR construct, e.g., if implementation is complex due to lacking sufficient funding, it would be coded to Inner Setting Domain: Available Resource: Funding.*  Some innovations seem quite simple, e.g., [SIMPLE INNOVATION], while others are quite complex, e.g., a year-long multidisciplinary program with a lot of moving parts: How complex is [INNOVATION]? | ***Note:*** *If respondents start to discuss the complexity of implementation (versus the complexity of the innovation), probe to identify the root causes of implementation complexity, in order to later code the appropriate CFIR construct, e.g., if implementation is complex due to lacking sufficient funding, it would be coded to Inner Setting Domain: Available Resource: Funding.*  Some innovations seem quite simple, e.g., [SIMPLE INNOVATION], while others are quite complex, e.g., a year-long multidisciplinary program with a lot of moving parts: Thinking back to before or during implementation: How complex did you think [INNOVATION] was? |
| G. Innovation Design | The innovation is well designed and packaged, including how it is assembled, bundled, and presented. | Consider the design of [INNOVATION] and the materials and resources that may be bundled with it: To what extent is [INNOVATION] well-designed?   - To what extent does [INNOVATION] include all necessary materials and resources, e.g., an implementation toolkit? | Consider the design of [INNOVATION] and the materials and resources that may be bundled with it:  Thinking back to before or during implementation:  To what extent did you think [INNOVATION] was well-designed?   - To what extent did [INNOVATION] include all necessary materials and resources, e.g., an implementation toolkit? |
| H. Innovation Cost | The innovation purchase and operating costs are affordable. | ***Note:*** *This construct captures the cost of the innovation, not the resources available in the Inner Setting to implement and deliver the innovation.*  How much does [INNOVATION] cost to purchase and/or operate?   - To what extent is [INNOVATION] affordable or expensive? | ***Note:*** *This construct captures the cost of the innovation, not the resources available in the Inner Setting to implement and deliver the innovation.*  Thinking back to before or during implementation:  How much did [INNOVATION] cost to purchase and/or operate?   - To what extent did you think [INNOVATION] was affordable or expensive? |

## Outer Setting Domain Questions

| **II. OUTER SETTING DOMAIN** | ***Outer Setting:*** The setting in which the Inner Setting exists, e.g., hospital system, school district, state. There may be multiple Outer Settings and/or multiple levels within the Outer Setting (e.g., community, system, state).  ***Project Outer Setting(s):*** [Document the actual Outer Setting in the project, e.g., type, location, and the boundary between the Outer Setting and the Inner Setting.] | | |
| --- | --- | --- | --- |
| **CFIR Construct Name** | **Construct Definition** *The degree to which:* | **Prospective Questions:** These questions will help assess determinants of Anticipated Implementation Outcomes [1]. | **Retrospective Questions:** These questions will help assess determinants of Actual Implementation Outcomes [1]. |
| A. Critical Incidents | Large-scale and/or unanticipated events disrupt implementation and/or delivery of the innovation. | Are there any recent unanticipated and/or large-scale events in [OUTER SETTING] that may influence implementing and/or delivering [INNOVATION]? If yes: How might this event influence implementation and/or delivery of [INNOVATION]? | Were there any recent unanticipated and/or large-scale events in [OUTER SETTING] that influenced implementing and/or delivering [INNOVATION]? If yes: How did this event influence implementation and/or delivery of [INNOVATION]? |
| B. Local Attitudes | Sociocultural values (e.g., shared responsibility in helping recipients) and beliefs (e.g., convictions about the worthiness of recipients) encourage the Outer Setting to support implementation and/or delivery of the innovation. | ***Note:*** *This construct was added to CFIR to address situations in which the Inner Setting requires explicit support, e.g., participation or funding, from the Outer Setting to implement and/or deliver the innovation. If the answer to the first question below is “none,” it’s unlikely this construct is relevant.*  What level of support (if any) is needed from [OUTER SETTING] to implement and/or deliver [INNOVATION]?   - What kinds of attitudes do people within [OUTER SETTING] have toward [INNOVATION RECIPIENTS]? - How do these attitudes encourage or discourage [OUTER SETTING] from supporting implementation and/or delivery of [INNOVATION]? | ***Note:*** *This construct was added to CFIR to address situations in which the Inner Setting requires explicit support, e.g., participation or funding, from the Outer Setting to implement and/or deliver the innovation. If the answer to the first question below is “none,” it’s unlikely this construct is relevant.*  What level of support (if any) was needed from [OUTER SETTING] to implement and/or deliver [INNOVATION]?   - What kinds of attitudes do people within [OUTER SETTING] have toward [INNOVATION RECIPIENTS]? - How did these attitudes encourage or discourage [OUTER SETTING] from supporting implementation and/or delivery of [INNOVATION]? |
| C. Local Conditions | Economic, environmental, political, and/or technological conditions enable the Outer Setting to support implementation and/or delivery of the innovation. | ***Note:*** *This construct was added to CFIR to address situations in which the Inner Setting requires explicit support, e.g., participation or funding, from the Outer Setting to implement and/or deliver the innovation. If the answer to the first question below is “none,” it’s unlikely this construct is relevant.*  What level of support (if any) is needed from [OUTER SETTING] to implement and/or deliver [INNOVATION]?   - What are the [CONDITIONS] in [OUTER SETTING]? - How do these [CONDITIONS] enable or hinder [OUTER SETTING] from supporting implementation and/or delivery of [INNOVATION]? | ***Note:*** *This construct was added to CFIR to address situations in which the Inner Setting requires explicit support, e.g., participation or funding, from the Outer Setting to implement and/or deliver the innovation. If the answer to the first question below is “none,” it’s unlikely this construct is relevant.*  What level of support (if any) was needed from [OUTER SETTING] to implement and/or deliver [INNOVATION]?   - What are the [CONDITIONS] in [OUTER SETTING]? - How did these [CONDITIONS] enable or hinder [OUTER SETTING] from supporting implementation and/or delivery of [INNOVATION]? |
| D. Partnerships & Connections | The Inner Setting is networked with external entities, including referral networks, academic affiliations, and professional organization networks. | To what extent does [INNER SETTING] have established relationships with entities in [OUTER SETTING]? How would you describe these relationships?  To what extent do you network or exchange information with colleagues outside of [INNER SETTING]? How would you describe this networking or information exchange? | Thinking back to before or during implementation:  To what extent did [INNER SETTING] have established relationships with entities in [OUTER SETTING]? How would you describe these relationships?  Thinking back to before or during implementation:  To what extent did you network or exchange information with colleagues outside of [INNER SETTING]? How would you describe this networking or information exchange? |
| E. Policies & Laws | Legislation, regulations, professional group guidelines and recommendations, or accreditation standards support implementation and/or delivery of the innovation. | What kinds of [OUTER SETTING] legislation, regulations, professional group recommendations, or accreditation standards might help or hinder implementation and/or delivery of [INNOVATION]? | What kinds of [OUTER SETTING] legislation, regulations, professional group recommendations, or accreditation standards helped or hindered implementation and/or delivery of [INNOVATION]? |
| F. Financing | Funding from external entities (e.g., grants, reimbursement) is available to implement and/or deliver the innovation. | What types of funding are needed from [OUTER SETTING] to implement and/or deliver [INNOVATION]? To what extent is this funding available? | What types of funding were needed from [OUTER SETTING] to implement and/or deliver [INNOVATION]? To what extent was this funding available? |
| G. External Pressure | External pressures drive implementation and/or delivery of the innovation.  *Use this construct to capture themes related to External Pressures that are not included in the subconstructs below.* | To what extent might pressures from [OUTER SETTING] influence implementation and/or delivery of [INNOVATION]? | To what extent did pressures from [OUTER SETTING] influence implementation and/or delivery of [INNOVATION]? |
| 1. Societal Pressure | Mass media campaigns, advocacy groups, or social movements or protests drive implementation and/or delivery of the innovation. | To what extent might societal pressures influence implementation and/or delivery of [INNOVATION]? | To what extent did societal pressures influence implementation and/or delivery of [INNOVATION]? |
| 2. Market Pressure | Competing with and/or imitating peer entities drives implementation and/or delivery of the innovation. | To what extent might competing with and/or imitating [INNER SETTING PEER ENTITIES] influence implementation and/or delivery of [INNOVATION]? | To what extent did competing with and/or imitating [INNER SETTING PEER ENTITIES] influence implementation and/or delivery of [INNOVATION]? |
| 3. Performance-Measurement Pressure | Quality or benchmarking metrics or established service goals drive implementation and/or delivery of the innovation. | To what extent might external performance measures/metrics influence implementation and/or delivery of [INNOVATION]? | To what extent did external performance measures/metrics influence implementation and/or delivery of [INNOVATION]? |

## Inner Setting Domain Questions

| **III. INNER SETTING DOMAIN** | ***Inner Setting:*** The setting in which the innovation is implemented, e.g., hospital, school, city. There may be multiple Inner Settings and/or multiple levels within the Inner Setting, e.g., unit, classroom, team.  ***Project Inner Setting(s):*** [Document the actual Inner Setting in the project, e.g., type, location, and the boundary between the Outer Setting and the Inner Setting.] | | |
| --- | --- | --- | --- |
| **CFIR Construct Name** | **Construct Definition** *The degree to which:* | **Prospective Questions:** These questions will help assess determinants of Anticipated Implementation Outcomes [1]. | **Retrospective Questions:** These questions will help assess determinants of Actual Implementation Outcomes [1]. |
|  | ***Note:*** *Constructs A – D exist in the Inner Setting regardless of implementation and/or delivery of the innovation, i.e., they are persistent general characteristics of the Inner Setting.*  *Retrospective questions for constructs A-D are written in present tense because these constructs represent persistent general characteristics of the Inner Setting.* | | |
| A. Structural Characteristics | Infrastructure components support functional performance of the Inner Setting.  *Use this construct to capture themes related to Structural Characteristics that are not included in the subconstructs below.* | Please describe how [STRUCTURAL CHARACTERISTICS] in [INNER SETTING] influence [INNER SETTING] functional performance. | Please describe how [STRUCTURAL CHARACTERISTICS] in [INNER SETTING] influence [INNER SETTING] functional performance. |
| 1. Physical Infrastructure | Layout and configuration of space and other tangible material features support functional performance of the Inner Setting. | Please describe how [PHYSICAL INFRASTRUCTURE] in [INNER SETTING] influences [INNER SETTING] functional performance. | Please describe how [PHYSICAL INFRASTRUCTURE] in [INNER SETTING] influences [INNER SETTING] functional performance. |
| 2. Information Technology Infrastructure | Technological systems for tele-communication, electronic documentation, and data storage, management, reporting, and analysis support functional performance of the Inner Setting. | Please describe how [TECHNOLOGICAL INFRASTRUCTURE] in [INNER SETTING] influences [INNER SETTING] functional performance. | Please describe how [TECHNOLOGICAL INFRASTRUCTURE] in [INNER SETTING] influences [INNER SETTING] functional performance. |
| 3. Work Infrastructure | Organization of tasks and responsibilities within and between individuals and teams, and general staffing levels, support functional performance of the Inner Setting. | Please describe how [WORK INFRASTRUCTURE] in [INNER SETTING] influences [INNER SETTING] functional performance.  Please describe how general staffing levels in [INNER SETTING] influences [INNER SETTING] functional performance. | Please describe how [WORK INFRASTRUCTURE] in [INNER SETTING] influences [INNER SETTING] functional performance.  Please describe how general staffing levels in [INNER SETTING] influences [INNER SETTING] functional performance. |
| B. Relational Connections | There are high quality formal and informal relationships, networks, and teams within and across Inner Setting boundaries (e.g., structural, professional). | How would you describe your working relationships with colleagues in your [INNER SETTING AREA]? With colleagues in other [INNER SETTING AREAS]?  To what extent do people work in teams? | How would you describe your working relationships with colleagues in your [INNER SETTING AREA]? With colleagues in other [INNER SETTING AREAS]?  To what extent do people work in teams? |
| C. Communications | There are high quality formal and informal information sharing practices within and across Inner Setting boundaries (e.g., structural, professional). | How do you typically hear or learn about things in [INNER SETTING]? What information would you like to have that is not usually shared? | How do you typically hear or learn about things in [INNER SETTING]? What information would you like to have that is not usually shared? |
| D. Culture | There are shared values, beliefs, and norms across the Inner Setting.  *Use this construct to capture themes related to Culture that are not included in the subconstructs below.* | How would you describe the culture of [INNER SETTING]? | How would you describe the culture of [INNER SETTING]? |
| 1. Human Equality-Centeredness | There are shared values, beliefs, and norms about the inherent equal worth and value of all human beings. | To what extent is there a culture of diversity, equity, and inclusion (DEI) in [INNER SETTING]? | To what extent is there a culture of diversity, equity, and inclusion (DEI) in [INNER SETTING]? |
| 2. Recipient-Centeredness | There are shared values, beliefs, and norms around caring, supporting, and addressing the needs and welfare of recipients. | ***Note:*** *“Marginalized” is used below to reference groups or individuals that are excluded due to systems of oppression, including but not limited to racism, sexism, heterosexism, cissexism, classism, ableism and sizeism.*  To what extent is there a [INNOVATION RECIPIENT]-Centered culture in [INNER SETTING]?  To what extent are [MARGINALIZED INNOVATION RECIPIENTS] centered in [INNER SETTING]? | ***Note:*** *“Marginalized” is used below to reference groups or individuals that are excluded due to systems of oppression, including but not limited to racism, sexism, heterosexism, cissexism, classism, ableism and sizeism.*  To what extent is there a [INNOVATION RECIPIENT]-Centered culture in [INNER SETTING]?  To what extent are [MARGINALIZED INNOVATION RECIPIENTS] centered in [INNER SETTING]? |
| 3. Deliverer-Centeredness | There are shared values, beliefs, and norms around caring, supporting, and addressing the needs and welfare of deliverers. | ***Note:*** *“Marginalized” is used below to reference groups or individuals that are excluded due to systems of oppression, including but not limited to racism, sexism, heterosexism, cissexism, classism, ableism and sizeism.*  To what extent is there a [INNOVATION DELIVERER]-Centered culture in [INNER SETTING]?  To what extent are [MARGINALIZED INNOVATION DELIVERERS] centered in [INNER SETTING]? | ***Note:*** *“Marginalized” is used below to reference groups or individuals that are excluded due to systems of oppression, including but not limited to racism, sexism, heterosexism, cissexism, classism, ableism and sizeism.*  To what extent is there a [INNOVATION DELIVERER]-Centered culture in [INNER SETTING]?  To what extent are [MARGINALIZED INNOVATION DELIVERERS] centered in [INNER SETTING]? |
| 4. Learning-Centeredness | There are shared values, beliefs, and norms around psychological safety, continual improvement, and using data to inform practice. | To what extent is there a culture of continual improvement, admitting to and learning from failure, and using data to inform practice in [INNER SETTING]? | To what extent is there a culture of continual improvement, admitting to and learning from failure, and using data to inform practice in [INNER SETTING]? |
|  | ***Note:*** *Constructs E – K are specific to the implementation and/or delivery of the innovation****.*** | | |
| E. Tension for Change | The current situation is intolerable and needs to change. | To what extent is [INNOVATION] needed in [INNER SETTING]? | To what extent was [INNOVATION] needed in [INNER SETTING]? |
| F. Compatibility | The innovation fits with workflows, systems, and processes. | To what extent does [INNOVATION] fit into existing work processes?   - What (if any) [INNOVATION] components or processes need to be changed to fit into existing [INNER SETTING] work processes? - What (if any) [INNER SETTING] work processes need to be changed to fit [INNOVATION]? | To what extent did [INNOVATION] fit into existing work processes?   - What (if any) [INNOVATION] components or processes needed to be changed to fit into existing [INNER SETTING] work processes? - What (if any) [INNER SETTING] work processes needed to be changed to fit [INNOVATION]? |
| G. Relative Priority | Implementing and delivering the innovation is important compared to other initiatives. | What is the priority of implementing and/or delivering [INNOVATION] compared to other initiatives? | What was the priority of implementing and/or delivering [INNOVATION] compared to other initiatives? |
| H. Incentive Systems | Tangible and/or intangible incentives and rewards and/or disincentives and punishments support implementation and delivery of the innovation. | What kinds of [INCENTIVES/DISINCENTIVES] in [INNER SETTING] may influence implementation and/or delivery of [INNOVATION]? | What kinds of [INCENTIVES/DISINCENTIVES] in [INNER SETTING] influenced implementation and/or delivery of [INNOVATION]? |
| I. Mission Alignment | Implementing and delivering the innovation is in line with the overarching commitment, purpose, or goals in the Inner Setting. | To what extent does implementing and/or delivering [INNOVATION] align with [INNER SETTING] mission and goals? | To what extent did implementing and/or delivering [INNOVATION] align with [INNER SETTING] mission and goals? |
| J. Available Resources | Resources are available to implement and deliver the innovation.  *Use this construct to capture themes related to Available Resources that are not included in the subconstructs below.* | To what extent are necessary resources available to implement and/or deliver [INNOVATION]? | To what extent were necessary resources available to implement and/or deliver [INNOVATION]? |
| 1. Funding | Funding is available to implement and deliver the innovation. | To what extent is necessary funding available to implement and/or deliver [INNOVATION]? | To what extent was necessary funding available to implement and/or deliver [INNOVATION]? |
| 2. Space | Physical space is available to implement and deliver the innovation. | To what extent is necessary space available to implement and/or deliver [INNOVATION]? | To what extent was necessary space available to implement and/or deliver [INNOVATION]? |
| 3. Materials & Equipment | Supplies are available to implement and deliver the innovation. | To what extent are necessary [MATERIALS AND EQUIPMENT] available to implement and/or deliver [INNOVATION]? | To what extent were necessary [MATERIALS AND EQUIPMENT] available to implement and/or deliver [INNOVATION]? |
| K. Access to Knowledge & Information | Guidance and/or training is accessible to implement and deliver the innovation. | What kinds of training and guidance are available to support implementation and/or delivery of [INNOVATION]? What information or training is missing? | What kinds of training and guidance were available to support implementation and/or delivery of [INNOVATION]? What information or training was missing? |

## Individuals Domain Questions

| **IV. INDIVIDUALS DOMAIN** | ***Individuals:*** The roles and characteristics of individuals. ***Note:*** *Roles may be internal or external to the Inner Setting.* | | |
| --- | --- | --- | --- |
| **ROLES SUBDOMAIN** | ***Project Roles:*** [Document the roles applicable to the project and their location in the Inner or Outer Setting.]  ***Note:*** *See the Characteristics Subdomain below for additional questions.* | | |
| **CFIR Construct Name** | **Construct Definition** | **Prospective Questions:** These questions will help assess determinants of Anticipated Implementation Outcomes [1]. | **Retrospective Questions:** These questions will help assess determinants of Actual Implementation Outcomes [1]. |
| A. High-level Leaders | Individuals with a high level of authority, including key decision-makers, executive leaders, or directors. | Who are [HIGH-LEVEL LEADERS]?  What is their role in implementation? | Who are [HIGH-LEVEL LEADERS]?  What was their role in implementation? |
| B. Mid-level Leaders | Individuals with a moderate level of authority, including leaders supervised by a high-level leader and who supervise others. | Who are [MID-LEVEL LEADERS]?  What is their role in implementation? | Who are [MID-LEVEL LEADERS]?  What was their role in implementation? |
| C. Opinion Leaders | Individuals with informal influence on the attitudes and behaviors of others. | Who are [OPINION LEADERS]?  What is their role in implementation? | Who are [OPINION LEADERS]?  What was their role in implementation? |
| D. Implementation Facilitators | Individuals with subject matter expertise who assist, coach, or support implementation. | Who are [IMPLEMENTATION FACILITATORS]?  What is their role in implementation? | Who are [IMPLEMENTATION FACILITATORS]?  What was their role in implementation? |
| E. Implementation Leads | Individuals who lead efforts to implement the innovation. | Who are [IMPLEMENTATION LEADS]?  What is their role in implementation? | Who are [IMPLEMENTATION LEADS]?  What was their role in implementation? |
| F. Implementation Team Members | Individuals who collaborate with and support the Implementation Leads to implement the innovation, ideally including Innovation Deliverers and Recipients. | Who are [IMPLEMENTATION TEAM MEMBERS]?  What is their role in implementation? | Who are [IMPLEMENTATION TEAM MEMBERS]?  What was their role in implementation? |
| G. Other Implementation Support | Individuals who support the Implementation Leads and/or Implementation Team Members to implement the innovation. | Who are [OTHER IMPLEMENTATION SUPPORT] individuals?  What is their role in implementation? | Who are [OTHER IMPLEMENTATION SUPPORT] individuals?  What was their role in implementation? |
| H. Innovation Deliverers | Individuals who are directly or indirectly delivering the innovation. | Who are [INNOVATION DELIVERERS]?  What is their role (if any) in implementation (versus delivery) of [INNOVATION]? | Who are [INNOVATION DELIVERERS]?  What was their role (if any) in implementation (versus delivery) of [INNOVATION]? |
| I. Innovation Recipients | Individuals who are directly or indirectly receiving the innovation. | Who are [INNOVATION RECIPIENTS]?  What is their role (if any) in implementation (vs. receipt) of [INNOVATION]? | Who are [INNOVATION RECIPIENTS]?  What was their role (if any) in implementation (vs. receipt) of [INNOVATION]? |
| **CHARACTERISTICS SUBDOMAIN** | ***Project Role Characteristics:*** [Document the characteristics applicable to the roles in the project based on the COM-B system [11] or role-specific theories. For example, theories related to:   - Behavior change, e.g., the Theoretical Domains Framework [12], [13], the Theory of Planned Behavior [14] or the Social Ecological Theory [15] may provide constructs more relevant for Innovation Recipients and Innovation Deliverers. - Facilitation [16], [17] and project management [18], [19] may provide constructs more relevant for Implementation Facilitators and Implementation Leads. - Leadership [16], [17] may provide constructs more relevant for High- and Mid-Level Leaders.   These role-specific constructs may be mapped to the broader COM-B constructs; for example, all 14 domains of the Theoretical Domains Framework (TDF) map to the COM-B system [11].]  ***Note:*** *See the Roles Subdomain above for additional questions. Some of the constructs in this domain may be more or less relevant depending on the associated role, e.g., most teams will want to assess Need related to Innovation Recipients, but that characteristic may not be relevant for other roles.* | | |
| **CFIR Construct Name** | **Construct Definition:**  *The degree to which:* | **Prospective Questions:** These questions will help assess determinants of Anticipated Implementation Outcomes [1]. | **Retrospective Questions:** These questions will help assess determinants of Actual Implementation Outcomes [1]. |
| A. Need | The individual(s) has deficits related to survival, well-being, or personal fulfillment, which will be addressed by implementation and/or delivery of the innovation. | To what extent do [ROLE] need [INNOVATION]? | Thinking back to the time before implementation, to what extent did [ROLE] need [INNOVATION]? |
| B. Capability | The individual(s) has interpersonal competence, knowledge, and skills to fulfill Role. | To what extent do [ROLE] have interpersonal competence, knowledge, and skills to [FULFILL ROLE]?  How well-suited are they to [FULFILL ROLE]? | To what extent did [ROLE] have interpersonal competence, knowledge, and skills to [FULFILL ROLE]?  How well-suited were they to [FULFILL ROLE]? |
| C. Opportunity | The individual(s) has availability, scope, and power to fulfill Role. | To what extent do [ROLE] have availability and/or protected time to [FULFILL ROLE]?  To what extent do [ROLE] have scope and power to [FULFILL ROLE]? | To what extent did [ROLE] have availability and/or protected time to [FULFILL ROLE]?  To what extent did [ROLE] have scope and power to [FULFILL ROLE]? |
| D. Motivation | The individual(s) is committed to fulfilling Role. | To what extent are [ROLE] committed to [FULFILLING ROLE]? | To what extent were [ROLE] committed to [FULFILLING ROLE]? |

## Implementation Process Domain Questions

| **V. IMPLEMENTATION PROCESS DOMAIN** | ***Implementation Process:*** The activities and strategies used to implement the innovation.  ***Project Implementation Process:*** [Document the implementation process framework [20] and/or activities and strategies [8], [9] being used to implement the innovation. Distinguish the implementation process used to implement the innovation (activities that end after implementation is complete) from the innovation (the “thing” that continues when implementation is complete) [2], [7], [10].] | | |
| --- | --- | --- | --- |
| **CFIR Construct Name** | **Construct Definition:** *The degree to which individuals:* | **Prospective Questions:** These questions will help assess determinants of Anticipated Implementation Outcomes [1].  ***Note:*** *Depending on the timing of the interview, some of the processes described in this domain may not yet have occurred and the associated questions may not be relevant.* | **Retrospective Questions:** These questions will help assess determinants of Actual Implementation Outcomes [1]. |
| A. Teaming | Join together, intentionally coordinating and collaborating on interdependent tasks, to implement the innovation. | To what extent (and how) is an implementation team being formed?  To what extent (and how) is the team intentionally coordinating and collaborating to complete implementation? | To what extent (and how) was an implementation team formed?  To what extent (and how) did the team intentionally coordinate and collaborate to complete tasks? |
| B. Assessing Needs | Collect information about priorities, preferences, and needs of people.  *Use this construct to capture themes related to Assessing Needs that are not included in the subconstructs below.* | To what extent (and how) is a needs assessment being conducted? | To what extent (and how) was a needs assessment conducted? |
| 1. Innovation Deliverers | Collect information about the priorities, preferences, and needs of deliverers to guide implementation and delivery of the innovation. | ***Note:*** *“Marginalized” is used below to reference groups or individuals that are excluded due to systems of oppression, including but not limited to racism, sexism, heterosexism, cissexism, classism, ableism and sizeism.*  To what extent (and how) is a needs assessment being conducted with [INNOVATION DELIVERERS]?  To what extent (and how) is a needs assessment being conducted with [MARGINALIZED INNOVATION DELIVERERS]? | ***Note:*** *“Marginalized” is used below to reference groups or individuals that are excluded due to systems of oppression, including but not limited to racism, sexism, heterosexism, cissexism, classism, ableism and sizeism.*  To what extent (and how) was a needs assessment conducted with [INNOVATION DELIVERERS]?  To what extent (and how) was a needs assessment conducted with [MARGINALIZED INNOVATION DELIVERERS]? |
| 2. Innovation Recipients | Collect information about the priorities, preferences, and needs of recipients to guide implementation and delivery of the innovation. | ***Note:*** *“Marginalized” is used below to reference groups or individuals that are excluded due to systems of oppression, including but not limited to racism, sexism, heterosexism, cissexism, classism, ableism and sizeism.*  To what extent (and how) is a needs assessment being conducted with [INNOVATION RECIPIENTS]?  To what extent (and how) is a needs assessment being conducted with [MARGINALIZED INNOVATION RECIPIENTS]? | ***Note:*** *“Marginalized” is used below to reference groups or individuals that are excluded due to systems of oppression, including but not limited to racism, sexism, heterosexism, cissexism, classism, ableism and sizeism.*  To what extent (and how) was a needs assessment conducted with [INNOVATION RECIPIENTS]?  To what extent (and how) was a needs assessment conducted with [MARGINALIZED INNOVATION RECIPIENTS]? |
| C. Assessing Context | Collect information to identify and appraise barriers and facilitators to implementation and delivery of the innovation. | To what extent (and how) are barriers and facilitators to implementation and delivery being assessed? | To what extent (and how) were barriers and facilitators to implementation and delivery assessed? |
| D. Planning | Identify roles and responsibilities, outline specific steps and milestones, and define goals and measures for implementation success in advance. | To what extent (and how) is an implementation plan being developed? What does the plan include?   - To what extent are roles and responsibilities being identified? - To what extent are specific steps and milestones being outlined? - To what extent are implementation goals being set? | To what extent (and how) was an implementation plan developed? What did the plan include?   - To what extent were roles and responsibilities identified? - To what extent were specific steps and milestones outlined? - To what extent were implementation goals set? |
| E. Tailoring Strategies | Choose and operationalize implementation strategies to address barriers, leverage facilitators, and fit context. | To what extent (and how) are implementation strategies being chosen and tailored to implement [INNOVATION]? | To what extent (and how) were implementation strategies chosen and tailored to implement [INNOVATION]? |
| F. Engaging | Attract and encourage participation in implementation and/or the innovation.  *Use this construct to capture themes related to Engaging that are not included in the subconstructs below.* | To what extent (and how) are strategies being used to engage key individuals in implementing and/or delivering [INNOVATION]? | To what extent (and how) were strategies used to engage key individuals in implementing and/or delivering [INNOVATION]? |
| 1. Innovation Deliverers | Attract and encourage deliverers to serve on the implementation team and/or to deliver the innovation. | ***Note:*** *“Marginalized” is used below to reference groups or individuals that are excluded due to systems of oppression, including but not limited to racism, sexism, heterosexism, cissexism, classism, ableism and sizeism.*  To what extent (and how) are strategies being used to engage [INNOVATION DELIVERERS] to serve on the implementation team? To deliver [INNOVATION]?  To what extent (and how) are strategies being used to engage [MARGINALIZED INNOVATION DELIVERERS] to serve on the implementation team? To deliver [INNOVATION]? | ***Note:*** *“Marginalized” is used below to reference groups or individuals that are excluded due to systems of oppression, including but not limited to racism, sexism, heterosexism, cissexism, classism, ableism and sizeism.*  To what extent (and how) were strategies used to engage [INNOVATION DELIVERERS] to serve on the implementation team? To deliver [INNOVATION]?  To what extent (and how) were strategies used to engage [MARGINALIZED INNOVATION DELIVERERS] to serve on the implementation team? To deliver [INNOVATION]? |
| 2. Innovation Recipients | Attract and encourage recipients to serve on the implementation team and/or participate in the innovation. | ***Note:*** *“Marginalized” is used below to reference groups or individuals that are excluded due to systems of oppression, including but not limited to racism, sexism, heterosexism, cissexism, classism, ableism and sizeism.*  To what extent (and how) are strategies being used to engage [INNOVATION RECIPIENTS] to serve on the implementation team? To participate in [INNOVATION]?  To what extent (and how) are strategies being used to engage [MARGINALIZED INNOVATION RECIPIENTS] to serve on the implementation team? To participate in [INNOVATION]? | ***Note:*** *“Marginalized” is used below to reference groups or individuals that are excluded due to systems of oppression, including but not limited to racism, sexism, heterosexism, cissexism, classism, ableism and sizeism.*  To what extent (and how) were strategies used to engage [INNOVATION RECIPIENTS] to serve on the implementation team? To participate in [INNOVATION]?  To what extent (and how) were strategies used to engage [MARGINALIZED INNOVATION RECIPIENTS] to serve on the implementation team? To participate in [INNOVATION]? |
| G. Doing | Implement in small steps, tests, or cycles of change to trial and cumulatively optimize delivery of the innovation. | ***Note:*** *This construct captures the process of trialing the innovation,* ***not*** *the need to trial nor the inherent trialability of the innovation.*  To what extent (and how) is [INNOVATION] being implemented using small steps, tests, or cycles of change? | ***Note:*** *This construct captures the process of trialing the innovation,* ***not*** *the need to trial nor the inherent trialability of the innovation.*  To what extent (and how) was [INNOVATION] implemented using small steps, tests, or cycles of change? |
| H. Reflecting & Evaluating | Collect and discuss quantitative and qualitative information about the success of implementation and the innovation.  *Use this construct to capture themes related to Reflecting & Evaluating that are not included in the subconstructs below.* | To what extent (and how) is the success (or effectiveness) of implementation and/or [INNOVATION] being evaluated? What information or data is being collected? Who is reviewing and discussing it? When or how frequently? | To what extent (and how) was the success (or effectiveness) of implementation and/or [INNOVATION] evaluated? What information or data was collected? Who reviewed and discussed it? When or how frequently? |
| 1. Implementation | Collect and discuss quantitative and qualitive information about the success of implementation. | To what extent (and how) is the success (or effectiveness) of implementation being evaluated? What information or data is being collected? Who is reviewing and discussing it? When or how frequently? | To what extent (and how) was the success (or effectiveness) of implementation evaluated? What information or data was collected? Who reviewed and discussed it? When or how frequently? |
| 2. Innovation | Collect and discuss quantitative and qualitative information about the success of the innovation. | To what extent (and how) is the success (or effectiveness) of [INNOVATION] being evaluated? What information or data is being collected? Who is reviewing and discussing it? When or how frequently? | To what extent (and how) was the success (or effectiveness) of [INNOVATION] evaluated? What information or data was collected? Who reviewed and discussed it? When or how frequently? |
| I. Adapting | Modify the innovation and/or the Inner Setting for optimal fit and integration into work processes. | ***Note:*** *This construct captures the process of adapting the innovation and/or Inner Setting,* ***not*** *the need to adapt nor the inherent adaptability of the innovation. Users may wish to add new subconstructs based on a reporting framework for adaptations, e.g., the* [*FRAME*](https://pubmed.ncbi.nlm.nih.gov/31171014/) [21]*.*  To what extent (and how) is [INNOVATION] being adapted to integrate into [INNER SETTING] work processes? To better meet [INNOVATION RECIPIENT] needs? To better meet [INNOVATION DELIVERER] needs?  To what extent (and how) will [INNER SETTING] work processes be adapted to integrate [INNOVATION]? | ***Note:*** *This construct captures the process of adapting the innovation and/or Inner Setting,* ***not*** *the need to adapt nor the inherent adaptability of the innovation. Users may wish to add new subconstructs based on a reporting framework for adaptations, e.g., the* [*FRAME*](https://pubmed.ncbi.nlm.nih.gov/31171014/) [21]*.*  To what extent (and how) was [INNOVATION] adapted to integrate into [INNER SETTING] work processes? To better meet [INNOVATION RECIPIENT] needs? To better meet [INNOVATION DELIVERER] needs?  To what extent (and how) were [INNER SETTING] work processes adapted to integrate [INNOVATION]? |

## Outcomes Addendum Questions

| **OUTCOMES ADDENDUM** | ***Note:*** *While outcomes are not a CFIR domain, it is often important to collect and analyze data related to implementation outcomes, which influence innovation outcomes.* | | |
| --- | --- | --- | --- |
| **Outcome Name** | **Outcome Definition:** | **Prospective Questions:** These questions will help assess Anticipated Implementation Outcomes [1]. | **Retrospective Questions:** The questions will help assess Actual Implementation Outcomes [1]. |
| Implementation Outcomes | Outcomes that capture the success or failure of implementation, i.e., implementation and delivery of the innovation in the Inner Setting. | | |
| Anticipated Implementation Outcomes | Predictions of future implementation success or failure, i.e., implementation outcomes that have not yet occurred.  ***Note:*** *These outcomes are forward-looking; constellations of CFIR determinants across domains predict these outcomes.* | | |
| Adoptability | The likelihood key decision-makers will decide to put the innovation in place/innovation deliverers will decide to deliver to innovation. | How do you define successful adoption of [INNOVATION]?  We define “successful adoption” as [OUTCOME DESCRIPTION]: With that in mind: Overall, from a scale of 1 to 10, where 1 is unsuccessful, and 10 is successful, how successful will [INNER SETTING] be adopting [INNOVATION]?   - Why did you choose that number? - What would it take to increase that number?   We define “equitable adoption” as [OUTCOME DESCRIPTION]: With that in mind: Overall, from a scale of 1 to 10, where 1 is unsuccessful, and 10 is successful, how successful will [INNER SETTING] be equitably adopting [INNOVATION]?   - Why did you choose that number? - What would it take to increase that number? | N/A (only assessed prospectively) |
| Implementability | The likelihood the innovation will be put in place or delivered. | How do you define successful implementation of [INNOVATION]?  We define “successful implementation” as [OUTCOME DESCRIPTION]: With that in mind: Overall, from a scale of 1 to 10, where 1 is unsuccessful, and 10 is successful, how successful will [INNER SETTING] be implementing [INNOVATION]?   - Why did you choose that number? - What would it take to increase that number?   We define “equitable implementation” as [OUTCOME DESCRIPTION]: With that in mind: Overall, from a scale of 1 to 10, where 1 is unsuccessful, and 10 is successful, how successful will [INNER SETTING] be equitably implementing [INNOVATION]?   - Why did you choose that number? - What would it take to increase that number? | N/A (only assessed prospectively) |
| Sustainability | The likelihood the innovation will be put in place or delivered over the long-term. | How do you define successful sustainment of [INNOVATION]?  We define “successful sustainment” as [OUTCOME DESCRIPTION] over the long-term: With that in mind: Overall, from a scale of 1 to 10, where 1 is unsuccessful, and 10 is successful, how successful will [INNER SETTING] be sustaining [INNOVATION]?   - Why did you choose that number? - What would it take to increase that number?   We define “equitable sustainment” as [OUTCOME DESCRIPTION] over the long-term: With that in mind: Overall, from a scale of 1 to 10, where 1 is unsuccessful, and 10 is successful, how successful will [INNER SETTING] be equitably sustaining [INNOVATION]?   - Why did you choose that number? - What would it take to increase that number? | N/A (only assessed prospectively) |
| Actual Implementation Outcomes | Observed (current or past) implementation success or failure, i.e., implementation outcomes that have occurred.  ***Note:*** *These outcomes are backward-looking; constellations of CFIR determinants across domains explain these outcomes* | | |
| Adoption | The extent key decision-makers decide to put the innovation in place/innovation deliverers decide to deliver the innovation. | N/A (only assessed retrospectively) | How do you define successful adoption of [INNOVATION]?  We define “successful adoption” as [OUTCOME DESCRIPTION]: With that in mind: Overall, from a scale of 1 to 10, where 1 is unsuccessful, and 10 is successful, how successful was [INNER SETTING] in adopting [INNOVATION]?   - Why did you choose that number? - What would it take to increase that number?   We define “equitable adoption” as [OUTCOME DESCRIPTION]: With that in mind: Overall, from a scale of 1 to 10, where 1 is unsuccessful, and 10 is successful, how successful was [INNER SETTING] equitably adopting [INNOVATION]?   - Why did you choose that number? - What would it take to increase that number? |
| Implementation | The extent the innovation is in place or being delivered. | N/A (only assessed retrospectively) | How do you define successful implementation of [INNOVATION]?  We define “successful implementation” as [OUTCOME DESCRIPTION]: With that in mind: Overall, from a scale of 1 to 10, where 1 is unsuccessful, and 10 is successful, how successful was [INNER SETTING] in implementing [INNOVATION]?   - Why did you choose that number? - What would it take to increase that number?   We define “equitable implementation” as [OUTCOME DESCRIPTION]: With that in mind: Overall, from a scale of 1 to 10, where 1 is unsuccessful, and 10 is successful, how successful was [INNER SETTING] equitably implementing [INNOVATION]?   - Why did you choose that number? - What would it take to increase that number? |
| Sustainment | The extent the innovation is in place or being delivered over the long-term. | N/A (only assessed retrospectively) | How do you define successful sustainment of [INNOVATION]?  We define “successful sustainment” as [OUTCOME DESCRIPTION] over the long-term: With that in mind: Overall, from a scale of 1 to 10, where 1 is unsuccessful, and 10 is successful, how successful was [INNER SETTING] in sustaining [INNOVATION]?   - Why did you choose that number? - What would it take to increase that number?   We define “equitable sustainment” as [OUTCOME DESCRIPTION] over the long-term: With that in mind: Overall, from a scale of 1 to 10, where 1 is unsuccessful, and 10 is successful, how successful was [INNER SETTING] equitably sustaining [INNOVATION]?   - Why did you choose that number? - What would it take to increase that number? |
| Innovation Outcomes | Outcomes capturing the success or failure of the innovation, based on the impact of the innovation on three important constituents: innovation recipients, innovation deliverers, and key decision-makers. | | |
| Innovation Effectiveness | The extent to which the Innovation is effective. | N/A | How do you define [INNOVATION] effectiveness or success?  We define “innovation effectiveness” as [OUTCOME DESCRIPTION]: With that in mind: Overall, from a scale of 1 to 10, where 1 is unsuccessful, and 10 is successful, how effective was [INNOVATION] in [INNER SETTING]?   - Why did you choose that number? - What would it take to increase that number?   We define “equitable innovation effectiveness” as [OUTCOME DESCRIPTION]: With that in mind: Overall, from a scale of 1 to 10, where 1 is unsuccessful, and 10 is successful, how equitably effective was [INNOVATION] in [INNER SETTING]?   - Why did you choose that number? - What would it take to increase that number? |
| Key Decision-Maker Impacts | The effect or influence the innovation has on key decision-makers and/or the system. | N/A | ***Note:*** *“Marginalized” is used below to reference groups or individuals that are excluded due to systems of oppression, including but not limited to racism, sexism, heterosexism, cissexism, classism, ableism and sizeism.*  What impact did [INNOVATION] have on [KEY DECISION-MAKERS]?  What impact did [INNOVATION] have on [MARGINALIZED KEY DECISION-MAKERS]? |
| Deliverer Impacts | The effect or influence the innovation has on deliverers. | N/A | ***Note:*** *“Marginalized” is used below to reference groups or individuals that are excluded due to systems of oppression, including but not limited to racism, sexism, heterosexism, cissexism, classism, ableism and sizeism.*  What impact did [INNOVATION] have on [INNOVATION DELIVERERS]?  What impact did [INNOVATION] have on [MARGINALIZED INNOVATION DELIVERERS]? |
| Recipient Impacts | The effect or influence the innovation has on recipients. | N/A | ***Note:*** *“Marginalized” is used below to reference groups or individuals that are excluded due to systems of oppression, including but not limited to racism, sexism, heterosexism, cissexism, classism, ableism and sizeism.*  What impact did [INNOVATION] have on [INNOVATION RECIPIENTS]?  What impact did [INNOVATION] have on [MARGINALIZED INNOVATION RECIPIENTS]? |

# Note: Should I use CFIR to collect data from innovation recipients (e.g., patients or students)?

When collecting data, researchers must be clear about the goal of data collection: 1) to predict and/or explain implementation outcomes based on implementation determinants (this is within the scope of CFIR); or 2) to predict and/or explain innovation outcomes based on innovation determinants (this is outside the scope of CFIR).

CFIR implementation determinants capture Inner Setting-level barriers and facilitators that predict and/or explain *implementation* *outcomes, i.e., the innovation being implemented and delivered as intended in the Inner Setting.* These determinants are denoted by the gray arrow in Figure 1 labeled *CFIR Implementation Determinants* in the CFIR User Guide manuscript*.* Data (qualitative and/or quantitative) on these determinants is best collected from individuals who have influence and/or power related to implementation (usually folks within the implementing setting); these typically include the key decision-makers and individuals implementing and/or delivering the innovation.

As a result, CFIR is not the appropriate framework to use when collecting data from recipients, unless recipients are also helping to implement and/or deliver the innovation in the Inner Setting. As reflected by Orlando et al., it is disappointing to note that “… while patients are part of the health-care organization and are essential to assessing *intervention [innovation]* effectiveness, they are a less influential component of *implementation* success in health-care settings than administrators and physicians” (emphases added) [22]. Although hospital systems are increasingly prioritizing patient-centered care, convening patient advisory boards, and involving patients in co-design of initiatives [23], [24], these efforts have not yet resulted in true power-sharing between innovation recipients and key decision-makers in the Inner Setting [25].

As a result, direct data collection from recipients does not usually inform implementation outcomes. Instead, data collection from key decision-makers and individuals implementing and/or delivering the innovation about *their* perceptions of recipients (e.g., recipient needs and characteristics), and how those perceptions encourage (or discourage) completing implementation, informs Implementation Outcomes. **Although CFIR is often not appropriate for use with recipients (because they rarely hold roles as key decision-makers or innovation implementers/deliverers in the Inner Setting), we hope that will change.** **Recipients *should* have greater influence, authority, and power in systems; the updated CFIR** **highlighted the importance of implementation teams *including* innovation recipients (and innovation deliverers) as members. When recipients serve in that role, we strongly encourage using CFIR to collect data about implementation determinants from them – because they are *also* implementation team members. Ultimately, equitable population impact is only possible when recipients are integrally involved in implementation and all** **key constituencies share power and make decisions together.**

In contrast to implementation determinants, innovation determinants capture recipient-level characteristics and/or experiences with the innovation that predict and/or explain *innovation outcomes.* These determinants are denoted by the gray arrow in Figure 1 labeled *Innovation Determinants* in the CFIR User Guide*.* Data (qualitative and/or quantitative) on these determinants is best collected from recipients. Innovation determinants include constructs or measures that are based on the theoretical framework underlying the innovation. For example, in a “small change” weight loss intervention designed for patients, innovation determinants included patient-level demographics, motivation and intention, and self-efficacy because the intervention was guided by social-psychological and goal-conflict theories [26]. This innovation was tested within a randomized clinical trial [27] and a subset of patient characteristics (innovation determinants) were explored in secondary analyses to help explain innovation outcomes [28], [29], [30], [31]. **CFIR was not designed to capture these theory-derived determinants of innovation outcomes, and adapting CFIR constructs for this purpose separates them from the underlying organizational theory.**

# References

[1] L. J. Damschroder, C. M. Reardon, M. A. Opra Widerquist, and J. Lowery, “Conceptualizing outcomes for use with the Consolidated Framework for Implementation Research (CFIR): the CFIR Outcomes Addendum,” *Implementation Sci*, vol. 17, no. 1, p. 7, Dec. 2022, doi: 10.1186/s13012-021-01181-5.

[2] G. M. Curran, “Implementation science made too simple: a teaching tool.,” *Implement Sci Commun*, vol. 1, p. 27, 2020, doi: 10.1186/s43058-020-00001-z.

[3] L. Albrecht, M. Archibald, D. Arseneau, and S. D. Scott, “Development of a checklist to assess the quality of reporting of knowledge translation interventions using the Workgroup for Intervention Development and Evaluation Research (WIDER) recommendations,” *Implementation Sci*, vol. 8, no. 1, p. 52, Dec. 2013, doi: 10.1186/1748-5908-8-52.

[4] M. Butler *et al.*, “AHRQ series on complex intervention systematic reviews—paper 3: adapting frameworks to develop protocols,” *Journal of Clinical Epidemiology*, vol. 90, pp. 19–27, Oct. 2017, doi: 10.1016/j.jclinepi.2017.06.013.

[5] The AIMD Writing/Working Group, P. Bragge, J. M. Grimshaw, C. Lokker, and H. Colquhoun, “AIMD - a validated, simplified framework of interventions to promote and integrate evidence into health practices, systems, and policies,” *BMC Med Res Methodol*, vol. 17, no. 1, p. 38, Dec. 2017, doi: 10.1186/s12874-017-0314-8.

[6] T. C. Hoffmann *et al.*, “Better reporting of interventions: template for intervention description and replication (TIDieR) checklist and guide,” *BMJ*, vol. 348, p. g1687, Mar. 2014, doi: 10.1136/bmj.g1687.

[7] R. Lengnick-Hall *et al.*, “Six practical recommendations for improved implementation outcomes reporting,” *Implementation Sci*, vol. 17, no. 1, p. 16, Dec. 2022, doi: 10.1186/s13012-021-01183-3.

[8] B. J. Powell *et al.*, “A compilation of strategies for implementing clinical innovations in health and mental health,” *Med Care Res Rev*, vol. 69, no. 2, pp. 123–57, Apr. 2012, doi: 10.1177/1077558711430690.

[9] B. J. Powell *et al.*, “A refined compilation of implementation strategies: results from the Expert Recommendations for Implementing Change (ERIC) project,” *Implementation Science*, vol. 10, no. 1, p. 21, 2015.

[10] H. Pinnock *et al.*, “Standards for Reporting Implementation Studies (StaRI) Statement,” *BMJ*, p. i6795, Mar. 2017, doi: 10.1136/bmj.i6795.

[11] S. Michie, M. M. van Stralen, and R. West, “The behaviour change wheel: A new method for characterising and designing behaviour change interventions,” *Implement Sci*, vol. 6, p. 42, Apr. 2011, doi: 10.1186/1748-5908-6-42.

[12] S. Michie *et al.*, “Making psychological theory useful for implementing evidence based practice: a consensus approach,” *Qual Saf Health Care*, vol. 14, no. 1, pp. 26–33, Feb. 2005, doi: 10.1136/qshc.2004.011155.

[13] J. Cane, D. O’Connor, and S. Michie, “Validation of the theoretical domains framework for use in behaviour change and implementation research,” *Implementation Sci*, vol. 7, no. 1, p. 37, Dec. 2012, doi: 10.1186/1748-5908-7-37.

[14] I. Ajzen, “The theory of planned behaviour: Reactions and reflections,” *Psychology & Health*, vol. 26, no. 9, pp. 1113–1127, Sep. 2011, doi: 10.1080/08870446.2011.613995.

[15] D. Stokols, “Translating Social Ecological Theory into Guidelines for Community Health Promotion,” *Am J Health Promot*, vol. 10, no. 4, pp. 282–298, Mar. 1996, doi: 10.4278/0890-1171-10.4.282.

[16] A. Metz, L. Louison, K. Burke, and C. Ward, “Implementation Support Practitioner Profile,” National Implementation Research Network, 2020. Accessed: Dec. 22, 2021. [Online]. Available: https://nirn.fpg.unc.edu/resources/implementation-support-practitioner-profile

[17] B. Albers, A. Metz, and K. Burke, “Implementation support practitioners – a proposal for consolidating a diverse evidence base,” *BMC Health Serv Res*, vol. 20, no. 1, p. 368, Dec. 2020, doi: 10.1186/s12913-020-05145-1.

[18] M. Barron and A. Barron, “Project Management Areas of Expertise,” in *Project Management*. [Online]. Available: https://cnx.org/contents/XpF315mY@11.6:_nDfs3nk@2/Project-Management-Areas-of-Expertise

[19] R. Müller and R. Turner, “Leadership competency profiles of successful project managers,” *International Journal of Project Management*, vol. 28, no. 5, pp. 437–448, Jul. 2010, doi: 10.1016/j.ijproman.2009.09.003.

[20] P. Nilsen, “Making sense of implementation theories, models and frameworks,” *Implementation science*, vol. 10, no. 1, Art. no. 1, 2015.

[21] S. W. Stirman, A. A. Baumann, and C. J. Miller, “The FRAME: an expanded framework for reporting adaptations and modifications to evidence-based interventions,” *Implementation Science*, vol. 14, no. 1, Art. no. 1, 2019.

[22] L. A. Orlando *et al.*, “Developing a common framework for evaluating the implementation of genomic medicine interventions in clinical care: the IGNITE Network’s Common Measures Working Group,” *Genet Med*, vol. 20, no. 6, pp. 655–663, Jun. 2018, doi: 10.1038/gim.2017.144.

[23] A. R. Lyon *et al.*, “The impact of inter-organizational alignment (IOA) on implementation outcomes: evaluating unique and shared organizational influences in education sector mental health,” *Implementation Science*, vol. 13, no. 1, Art. no. 1, 2018.

[24] A. R. Dopp, K. E. Parisi, S. A. Munson, and A. R. Lyon, “Integrating implementation and user-centred design strategies to enhance the impact of health services: protocol from a concept mapping study,” *Health Res Policy Sys*, vol. 17, no. 1, p. 1, Dec. 2019, doi: 10.1186/s12961-018-0403-0.

[25] J. Trofino, “Power sharing. A transformational strategy for nurse retention, effectiveness, and extra effort,” *Nurs Leadersh Forum*, vol. 8, no. 2, pp. 64–71, 2003.

[26] L. D. Lutes *et al.*, “A randomized trial of a small changes approach for weight loss in veterans: Design, rationale, and baseline characteristics of the ASPIRE-VA trial,” *Contemporary Clinical Trials*, vol. 34, no. 1, pp. 161–172, Jan. 2013, doi: 10.1016/j.cct.2012.09.007.

[27] L. J. Damschroder *et al.*, “Small-Changes Obesity Treatment Among Veterans,” *American Journal of Preventive Medicine*, vol. 47, no. 5, pp. 541–553, Nov. 2014, doi: 10.1016/j.amepre.2014.06.016.

[28] R. M. Masheb *et al.*, “Weight loss outcomes in patients with pain: Weight Loss and Pain,” *Obesity*, vol. 23, no. 9, pp. 1778–1784, Sep. 2015, doi: 10.1002/oby.21160.

[29] R. M. Masheb *et al.*, “High-frequency binge eating predicts weight gain among veterans receiving behavioral weight loss treatments: High-Frequency Binge Eating and Weight Gain,” *Obesity*, vol. 23, no. 1, pp. 54–61, Jan. 2015, doi: 10.1002/oby.20931.

[30] V. Vimalananda *et al.*, “Weight loss among women and men in the ASPIRE-VA behavioral weight loss intervention trial: Sex-Specific Weight Loss Results in ASPIRE-VA,” *Obesity*, vol. 24, no. 9, pp. 1884–1891, Sep. 2016, doi: 10.1002/oby.21574.

[31] C. A. Janney *et al.*, “Mental health and behavioral weight loss: 24-month outcomes in Veterans,” *Journal of Affective Disorders*, vol. 215, pp. 197–204, Jun. 2017, doi: 10.1016/j.jad.2017.03.003.
