# Supplementary material for: The Consolidated Framework for Implementation Research (CFIR) User Guide: a five-step guide for conducting implementation research using the framework
Source: Implement Sci. 2025 Aug 16;20:39. doi: 10.1186/s13012-025-01450-7 (PMC12357348; doi:10.1186/s13012-025-01450-7)
Supplement: Supplementary file 3 — Additional file 3: Inner Setting Memo Template. [file 13012_2025_1450_MOESM3_ESM.docx]

[Inner Setting] Memo Template

# Introduction

This document facilitates aggregating, summarizing, and rating data for each construct at the Inner Setting (i.e., unit of analysis) level.

*Note: If conducting rapid qualitative analysis, data is aggregated during coding in the CFIR Construct x Inner Setting Matrix Template (Additional File 5 in the CFIR User Guide Manuscript) via a building approach as interviews progress. See previous publication* [1] *(Nevedal et al. 2021) and presentation* [2] *for more detail on completing rapid qualitative analysis using CFIR (and how it compares to the in-depth qualitative approach).*

Aggregating data facilitates summarizing and rating data for each construct; ratings are especially useful when there are at least three Inner Settings and there is interest in comparing constructs across Inner Settings based on implementation outcomes. Detailed rating guidelines are provided in Additional File 4 of the CFIR User Guide Manuscript. As with coding, we recommend using a consensus-based approach to finalize ratings. Depending on the project, it may not be helpful to rate the data, or users may wish to collapse ratings into a binary, e.g., barrier vs. facilitator, and only complete the valence (+ vs -) component of rating. See the CFIR User Guide Manuscript for more information.

*Note: This document is the first iteration of the Inner Setting Memo Template with updated CFIR constructs; please provide feedback regarding this template via www.cfirguide.org in order for us to improve it!*

| Analysts | Participants |
| --- | --- |
| Primary: [Primary Analyst Name]  Secondary: [Secondary Analyst Name] | Participant [ID Number]: Role  Participant [ID Number]: Role  Participant [ID Number]: Role |

# Inner Setting Summary

*[Draft high-level summary of the Inner Setting]*

# Innovation Domain

## Innovation Source

Overall Rating: __ (Primary Analyst: __; Secondary Analyst: __)

Summary:

*[Draft summary of data at Inner Setting Level, a summary of the data from all of the participants]*

Rationale:

*[Draft rationale for overall rating]*

Data:

Participant [ID Number] (Primary Analyst: __; Secondary Analyst: __)

*[Copy coded data from Participant [ID Number]]*

Participant [ID Number] (Primary Analyst: __; Secondary Analyst: __)

*[Copy coded data from Participant [ID Number]]*

Participant [ID Number] (Primary Analyst: __; Secondary Analyst: __)

*[Copy coded data from Participant [ID Number]]*

Participant [ID Number] (Primary Analyst: __; Secondary Analyst: __)

*[Copy coded data from Participant [ID Number]]*

## Innovation Evidence-Base

Overall Rating: __ (Primary Analyst: __; Secondary Analyst: __)

Summary:

Rationale:

Data:

## Innovation Relative Advantage

Overall Rating: __ (Primary Analyst: __; Secondary Analyst: __)

Summary:

Rationale:

Data:

## Innovation Adaptability

Overall Rating: __ (Primary Analyst: __; Secondary Analyst: __)

Summary:

Rationale:

Data:

## Innovation Trialability

Overall Rating: __ (Primary Analyst: __; Secondary Analyst: __)

Summary:

Rationale:

Data:

## Innovation Complexity

Overall Rating: __ (Primary Analyst: __; Secondary Analyst: __)

Summary:

Rationale:

Data:

## Innovation Design

Overall Rating: __ (Primary Analyst: __; Secondary Analyst: __)

Summary:

Rationale:

Data:

## Innovation Cost

Overall Rating: __ (Primary Analyst: __; Secondary Analyst: __)

Summary:

Rationale:

Data:

# Outer Setting Domain

## Critical Incidents

Overall Rating: __ (Primary Analyst: __; Secondary Analyst: __)

Summary:

Rationale:

Data:

## Local Attitudes

Overall Rating: __ (Primary Analyst: __; Secondary Analyst: __)

Summary:

Rationale:

Data:

## Local Conditions

Overall Rating: __ (Primary Analyst: __; Secondary Analyst: __)

Summary:

Rationale:

Data:

## Partnerships & Connections

Overall Rating: __ (Primary Analyst: __; Secondary Analyst: __)

Summary:

Rationale:

Data:

## Policies & Laws

Overall Rating: __ (Primary Analyst: __; Secondary Analyst: __)

Summary:

Rationale:

Data:

## Financing

Overall Rating: __ (Primary Analyst: __; Secondary Analyst: __)

Summary:

Rationale:

Data:

## External Pressure

### Societal Pressure

Overall Rating: __ (Primary Analyst: __; Secondary Analyst: __)

Summary:

Rationale:

Data:

### Market Pressure

Overall Rating: __ (Primary Analyst: __; Secondary Analyst: __)

Summary:

Rationale:

Data:

### Performance-Measurement Pressure

Overall Rating: __ (Primary Analyst: __; Secondary Analyst: __)

Summary:

Rationale:

Data:

# Inner Setting Domain

## Structural Characteristics

### Physical Infrastructure

Overall Rating: __ (Primary Analyst: __; Secondary Analyst: __)

Summary:

Rationale:

Data:

### Information Technology Infrastructure

Overall Rating: __ (Primary Analyst: __; Secondary Analyst: __)

Summary:

Rationale:

Data:

### Work Infrastructure

Overall Rating: __ (Primary Analyst: __; Secondary Analyst: __)

Summary:

Rationale:

Data:

## Relational Connections

Overall Rating: __ (Primary Analyst: __; Secondary Analyst: __)

Summary:

Rationale:

Data:

## Communications

Overall Rating: __ (Primary Analyst: __; Secondary Analyst: __)

Summary:

Rationale:

Data:

## Culture

### Human Equality-Centeredness

Overall Rating: __ (Primary Analyst: __; Secondary Analyst: __)

Summary:

Rationale:

Data:

### Recipient-Centeredness

Overall Rating: __ (Primary Analyst: __; Secondary Analyst: __)

Summary:

Rationale:

Data:

### Deliverer-Centeredness

Overall Rating: __ (Primary Analyst: __; Secondary Analyst: __)

Summary:

Rationale:

Data:

### Learning-Centeredness

Overall Rating: __ (Primary Analyst: __; Secondary Analyst: __)

Summary:

Rationale:

Data:

## Tension for Change

Overall Rating: __ (Primary Analyst: __; Secondary Analyst: __)

Summary:

Rationale:

Data:

## Compatibility

Overall Rating: __ (Primary Analyst: __; Secondary Analyst: __)

Summary:

Rationale:

Data:

## Relative Priority

Overall Rating: __ (Primary Analyst: __; Secondary Analyst: __)

Summary:

Rationale:

Data:

## Incentive Systems

Overall Rating: __ (Primary Analyst: __; Secondary Analyst: __)

Summary:

Rationale:

Data:

## Mission Alignment

Overall Rating: __ (Primary Analyst: __; Secondary Analyst: __)

Summary:

Rationale:

Data:

## Available Resources

### Funding

Overall Rating: __ (Primary Analyst: __; Secondary Analyst: __)

Summary:

Rationale:

Data:

### Space

Overall Rating: __ (Primary Analyst: __; Secondary Analyst: __)

Summary:

Rationale:

Data:

### Materials & Equipment

Overall Rating: __ (Primary Analyst: __; Secondary Analyst: __)

Summary:

Rationale:

Data:

## Access to Knowledge & Information

Overall Rating: __ (Primary Analyst: __; Secondary Analyst: __)

Summary:

Rationale:

Data:

# Individuals: Roles & Characteristics

## High-level Leaders

### Need

Overall Rating: __ (Primary Analyst: __; Secondary Analyst: __)

Summary:

Rationale:

Data:

### Capability

Overall Rating: __ (Primary Analyst: __; Secondary Analyst: __)

Summary:

Rationale:

Data:

### Opportunity

Overall Rating: __ (Primary Analyst: __; Secondary Analyst: __)

Summary:

Rationale:

Data:

### Motivation

Overall Rating: __ (Primary Analyst: __; Secondary Analyst: __)

Summary:

Rationale:

Data:

## Mid-level Leaders

### Need

Overall Rating: __ (Primary Analyst: __; Secondary Analyst: __)

Summary:

Rationale:

Data:

### Capability

Overall Rating: __ (Primary Analyst: __; Secondary Analyst: __)

Summary:

Rationale:

Data:

### Opportunity

Overall Rating: __ (Primary Analyst: __; Secondary Analyst: __)

Summary:

Rationale:

Data:

### Motivation

Overall Rating: __ (Primary Analyst: __; Secondary Analyst: __)

Summary:

Rationale:

Data:

## Opinion Leaders

### Need

Overall Rating: __ (Primary Analyst: __; Secondary Analyst: __)

Summary:

Rationale:

Data:

### Capability

Overall Rating: __ (Primary Analyst: __; Secondary Analyst: __)

Summary:

Rationale:

Data:

### Opportunity

Overall Rating: __ (Primary Analyst: __; Secondary Analyst: __)

Summary:

Rationale:

Data:

### Motivation

Overall Rating: __ (Primary Analyst: __; Secondary Analyst: __)

Summary:

Rationale:

Data:

## Implementation Facilitators

### Need

Overall Rating: __ (Primary Analyst: __; Secondary Analyst: __)

Summary:

Rationale:

Data:

### Capability

Overall Rating: __ (Primary Analyst: __; Secondary Analyst: __)

Summary:

Rationale:

Data:

### Opportunity

Overall Rating: __ (Primary Analyst: __; Secondary Analyst: __)

Summary:

Rationale:

Data:

### Motivation

Overall Rating: __ (Primary Analyst: __; Secondary Analyst: __)

Summary:

Rationale:

Data:

## Implementation Leads

### Need

Overall Rating: __ (Primary Analyst: __; Secondary Analyst: __)

Summary:

Rationale:

Data:

### Capability

Overall Rating: __ (Primary Analyst: __; Secondary Analyst: __)

Summary:

Rationale:

Data:

### Opportunity

Overall Rating: __ (Primary Analyst: __; Secondary Analyst: __)

Summary:

Rationale:

Data:

### Motivation

Overall Rating: __ (Primary Analyst: __; Secondary Analyst: __)

Summary:

Rationale:

Data:

## Implementation Team Members

### Need

Overall Rating: __ (Primary Analyst: __; Secondary Analyst: __)

Summary:

Rationale:

Data:

### Capability

Overall Rating: __ (Primary Analyst: __; Secondary Analyst: __)

Summary:

Rationale:

Data:

### Opportunity

Overall Rating: __ (Primary Analyst: __; Secondary Analyst: __)

Summary:

Rationale:

Data:

### Motivation

Overall Rating: __ (Primary Analyst: __; Secondary Analyst: __)

Summary:

Rationale:

Data:

## Other Implementation Support

### Need

Overall Rating: __ (Primary Analyst: __; Secondary Analyst: __)

Summary:

Rationale:

Data:

### Capability

Overall Rating: __ (Primary Analyst: __; Secondary Analyst: __)

Summary:

Rationale:

Data:

### Opportunity

Overall Rating: __ (Primary Analyst: __; Secondary Analyst: __)

Summary:

Rationale:

Data:

### Motivation

Overall Rating: __ (Primary Analyst: __; Secondary Analyst: __)

Summary:

Rationale:

Data:

## Innovation Deliverers

### Need

Overall Rating: __ (Primary Analyst: __; Secondary Analyst: __)

Summary:

Rationale:

Data:

### Capability

Overall Rating: __ (Primary Analyst: __; Secondary Analyst: __)

Summary:

Rationale:

Data:

### Opportunity

Overall Rating: __ (Primary Analyst: __; Secondary Analyst: __)

Summary:

Rationale:

Data:

### Motivation

Overall Rating: __ (Primary Analyst: __; Secondary Analyst: __)

Summary:

Rationale:

Data:

## Innovation Recipients

### Need

Overall Rating: __ (Primary Analyst: __; Secondary Analyst: __)

Summary:

Rationale:

Data:

### Capability

Overall Rating: __ (Primary Analyst: __; Secondary Analyst: __)

Summary:

Rationale:

Data:

### Opportunity

Overall Rating: __ (Primary Analyst: __; Secondary Analyst: __)

Summary:

Rationale:

Data:

### Motivation

Overall Rating: __ (Primary Analyst: __; Secondary Analyst: __)

Summary:

Rationale:

Data:

# Implementation Process

## Teaming

Overall Rating: __ (Primary Analyst: __; Secondary Analyst: __)

Summary:

Rationale:

Data:

## Assessing Needs

### Innovation Deliverers

Overall Rating: __ (Primary Analyst: __; Secondary Analyst: __)

Summary:

Rationale:

Data:

### Innovation Recipients

Overall Rating: __ (Primary Analyst: __; Secondary Analyst: __)

Summary:

Rationale:

Data:

## Assessing Context

Overall Rating: __ (Primary Analyst: __; Secondary Analyst: __)

Summary:

Rationale:

Data:

## Planning

Overall Rating: __ (Primary Analyst: __; Secondary Analyst: __)

Summary:

Rationale:

Data:

## Tailoring Strategies

Overall Rating: __ (Primary Analyst: __; Secondary Analyst: __)

Summary:

Rationale:

Data:

## Engaging

### Innovation Deliverers

Overall Rating: __ (Primary Analyst: __; Secondary Analyst: __)

Summary:

Rationale:

Data:

### Innovation Recipients

Overall Rating: __ (Primary Analyst: __; Secondary Analyst: __)

Summary:

Rationale:

Data:

## Doing

Overall Rating: __ (Primary Analyst: __; Secondary Analyst: __)

Summary:

Rationale:

Data:

## Reflecting & Evaluating

### Implementation

Overall Rating: __ (Primary Analyst: __; Secondary Analyst: __)

Summary:

Rationale:

Data:

### Innovation

Overall Rating: __ (Primary Analyst: __; Secondary Analyst: __)

Summary:

Rationale:

Data:

## Adapting

Overall Rating: __ (Primary Analyst: __; Secondary Analyst: __)

Summary:

Rationale:

Data:

# References

[1] A. L. Nevedal *et al.*, “Rapid versus traditional qualitative analysis using the Consolidated Framework for Implementation Research (CFIR),” *Implement. Sci.*, vol. 16, no. 1, p. 67, Jul. 2021, doi: 10.1186/s13012-021-01111-5.

[2] C. M. Reardon and A. L. Nevedal, “Rapid Analysis Using the Consolidated Framework for Implementation Research (CFIR): A Methods Cyberseminar,” HSR&D Cyberseminars, Oct. 14, 2021. Accessed: Mar. 03, 2024. [Online]. Available: https://www.hsrd.research.va.gov/for_researchers/cyber_seminars/archives/video_archive.cfm?SessionID=4044&Seriesid=113
