# Supplementary material for: The Consolidated Framework for Implementation Research (CFIR) User Guide: a five-step guide for conducting implementation research using the framework
Source: Implement Sci. 2025 Aug 16;20:39. doi: 10.1186/s13012-025-01450-7 (PMC12357348; doi:10.1186/s13012-025-01450-7)
Supplement: Supplementary file 6 — Additional file 6: CFIR Implementation Research Worksheet. [file 13012_2025_1450_MOESM6_ESM.docx]

# CFIR Implementation Research Planning Worksheet

## Introduction

The overarching aim of CFIR is to predict or explain barriers and facilitators (i.e., the determinants or independent variables) to implementation success at the Inner Setting level (i.e., the outcome or dependent variable). This worksheet follows the steps outlined in the CFIR User Guide manuscript.

CFIR was not designed to guide development of innovations nor to specify the process of implementation (see FAQs 4 & 5 in the CFIR User Guide manuscript for more information).

CFIR may be useful when your project meets the following criteria:

- Your research question includes predicting and/or explaining implementation outcomes based on implementation determinants
- The unit of analysis is a defined Inner Setting that will be implementing and delivering the innovation, e.g., hospital, school, city
- The team has a methodologist and/or analyst with experience in implementation science methods and/or using CFIR

## Step 1: Study Design

### 1A: Define Research Question and Implementation Outcome

| **Research Question**  What is your research question?  Are you prospectively assessing determinants of anticipated implementation outcomes (outcomes that have not yet occurred) and/or retrospectively assessing determinants of actual implementation outcomes (outcomes that have occurred)? | **Define your research question:** |
| --- | --- |
| **Implementation Outcome**  What is your implementation outcome?  *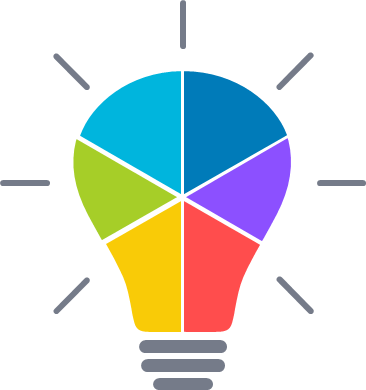When using CFIR, implementation refers to implementation by the Inner Setting (e.g., hospital), not by the recipient (e.g., patient).* | **Define your implementation outcome:** |

### 1B: Define CFIR (Implementation Determinant) Domains

| **Innovation**  What is the **innovation** being implemented and evaluated?   - What are its components and features [1], [2]? - What is the boundary between the innovation and the process or strategy being used to implement the innovation?   What is the (intended) **innovation outcome** for:   - Innovation Recipients - Innovation Deliverers - High-Level Leaders/Key Decision-Makers | **Define the innovation:**  **Define innovation outcomes for:**   - Innovation Recipients: - Innovation Deliverers: - High-Level Leaders/Key Decision-Makers: |
| --- | --- |
| **Individuals: Roles & Characteristics**  Who are the **individuals** involved with implementing, delivering, and/or receiving the innovation?   - What are their **roles**? - What are their **characteristics**? | **Define the individuals and their roles & characteristics:** |
| **Inner Setting & Outer Setting**  **Where** is implementation and delivery of the innovation occurring?   - What is the boundary between the Inner Setting (the unit of analysis and location where the innovation is being implemented) and the Outer Setting (the area outside of the Inner Setting)? | **Define the inner & outer setting:** |
| **Implementation Process**  What is the **implementation process**?   - Is implementation being guided by a specific implementation strategy or process model [3] (e.g., Knowledge to Action Framework [4], Getting To Outcomes [5], or Getting To Implementation [6])? - What are its components and features? - What is the boundary between the innovation and the process or strategy being used to implement the innovation? | **Define the implementation process:** |

## Step 2: Data Collection

### 2A: Determine Data Collection Approach

| **Data Collection Approach**  Considering your research question and the following criteria, select the best data collection approach(es) for your project:   - Participant Burden - Analyst Hours & CFIR Expertise - Transcription Delay & Cost - Level of Detail - Rigor   See Table 2 in the CFIR User Guide manuscript for more information. | **List the data collection approach(es), e.g., interviews, surveys, you will use and the rationale for this approach:** |
| --- | --- |

### 2B: Develop Data Collection Instrument

| **Construct Selection**  After defining the research question, each construct should be assessed for its likelihood of 1) being a potential barrier or facilitator to the innovation being implemented or 2) having sufficient variation across the units of analysis (i.e., the Inner Settings). Identifying relevant constructs may be completed by:   - Conducting informal interviews, surveys, or group deliberations with project team members, operational partners, and/or individuals with direct knowledge of the innovation and/or implementing setting - Reviewing and/or synthesizing the existing literature and implementation theories, models, and frameworks   *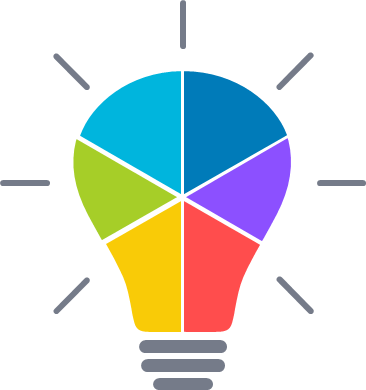*  *Include open-ended, non-construct specific questions to capture determinants not included in CFIR!*  Examples of questions are available in Additional File 1 of the CFIR User Guide manuscript. Following development of your data collection instrument, we recommend piloting the instrument.  *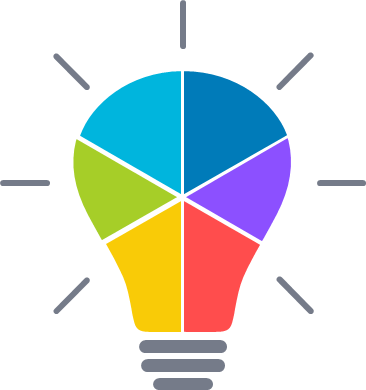*  *We do not recommend including a question about every CFIR construct in data collection instruments.* | **List the** [**constructs**](https://cfirguide.org/constructs) **you will include in your data collection instrument as well as the rationale for their inclusion:** |
| --- | --- |

### 2C: Develop Sampling Strategy

| **Sampling Strategy**  Although CFIR is used to collect data from individuals, information from individual respondents is aggregated to understand constructs at the Inner Setting (i.e., unit of analysis) level.  As a result, users must first identify Inner Settings, and then the individuals in each Inner Setting, to develop their sampling strategy. See the CFIR User Guide manuscript for more information on purposeful sampling [7] of Inner Settings and individuals.  *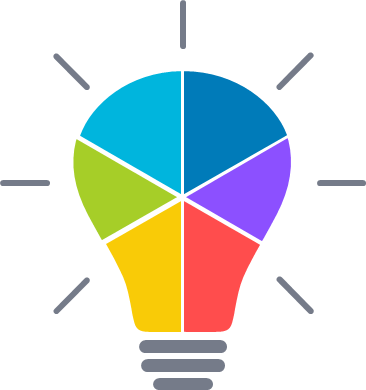CFIR should only be used to collect data from individuals who have influence and/or power related to implementation and/or delivery of the innovation in the Inner Setting.*  Innovation recipients, e.g., patients or students, are only appropriate to include in the sample of an implementation research study if they have insights into barriers or facilitators to implementation of the innovation in the Inner Setting, e.g., they were part of the implementation team (*see FAQ 10 in the CFIR User Guide manuscript for more information).* | **List the inner settings you will include in your sample as well as the rationale for their inclusion:**  **List the individuals you will include in your sample as well as the rationale for their inclusion:** |
| --- | --- |

### 2D: Conduct Data Collection

| **Data Collection**  It is outside the scope of this guide to offer specific direction around collecting data, and there are many high-quality sources on conducting interviews [8], [9] and focus groups [10], completing observations [11], [12], [13] and ethnographies [14], [15], obtaining periodic reflections [16], gathering archival data [17], and administering surveys [18].  *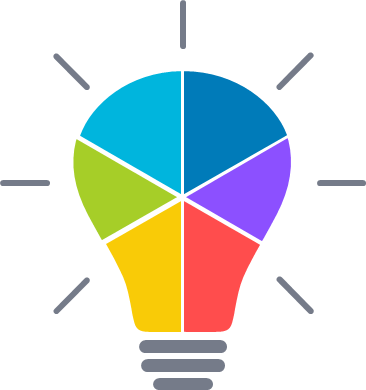To identify constructs that distinguish between implementation success and failure, data must be collected on both determinants and outcomes.* | **How will you prepare to conduct data collection, e.g., review materials, practice using the instrument?** |
| --- | --- |

## Step 3: Data Analysis

### 3A: Determine Data Analysis Approach

| **Data Analysis Approach**  Considering your research question, the type of data you collected (i.e., qualitative or quantitative), and the following criteria, select the best data analysis approach(es) for your project:   - Analyst Hours & CFIR Expertise - Transcription Delay & Cost - Level of Detail - Rigor   See Table 2 in the CFIR User Guide manuscript for more information. | **List the data analysis approach(es), e.g., qualitative, quantitative, you will use and the rationale for this approach:** |
| --- | --- |

### 3B: Conduct Data Analysis

| **Data Analysis**  **Coding Qualitative Data:**  CFIR provides the initial structure for a qualitative codebook, and detailed coding guidelines for each construct are provided in Additional File 2 in the CFIR User Guide Manuscript.  After coding, data should be aggregated by unit of analysis, i.e., Inner Setting, and CFIR construct; Additional Files 3 and 5 in the CFIR User Guide manuscript can help with aggregating and summarizing data.  **Rating Qualitative Data:**  Ratings are especially useful when there are at least three Inner Settings and there is interest in comparing constructs across Inner Settings based on implementation outcomes. Detailed rating guidelines are provided in Additional File 4 in the CFIR User Guide manuscript.  *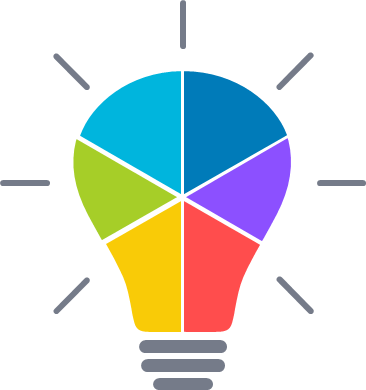We recommend using a consensus-based and iterative process* [19], [20] *including at least two analysts when coding and rating data.* | **How will you prepare to conduct data analysis, e.g., review materials, operationalize the coding guidelines?** |
| --- | --- |

## Step 4: Data Interpretation

### 4A: Align Implementation Determinants & Outcomes

| **Implementation Determinant & Outcome Alignment**  To identify constructs that distinguish between Inner Settings (i.e., unit of analysis) with high and low implementation success, integrate data on implementation determinants and outcomes (see the CFIR User Guide manuscript for additional information and Additional File 5 for a CFIR Construct x Inner Setting Matrix Template). | **How will you align implementation determinants and outcomes?** |
| --- | --- |

### 4B: Determine Data Interpretation Approach

| **Data Interpretation Approach**  Considering the research question, the data you collected, and sample size, select the best data interpretation approach(es) for your project:   - Visual Comparison - Correlational Analysis or Regression Modeling - Configurational Comparative Methods (CCMs) - Other?   See the CFIR User Guide Manuscript for more information on each approach. | **List the data interpretation approach(es) you will use and the rationale for this approach:** |
| --- | --- |

## Step 5: Knowledge Dissemination

### 5A: Determine Knowledge Dissemination Approach

| **Knowledge Dissemination Approach**  Planning dissemination early helps to ensure that you collect data that is meaningful to the audiences of interest. Considering your research question and audience(s), select the best data visualization approach(es) for your project:   - Traditional narrative that includes descriptions of the findings and representative quotes - Matrix of key barriers and facilitators with exemplar quotes - Table of frequencies of various barriers and facilitators - “Joint display” of the qualitative and quantitative results [21] - Implementation research logic model that highlights key barriers and their associations with outcomes and strategies [22] | **Who needs to see the results?**  **What presentation of the data would be most impactful for this audience(s)?** |
| --- | --- |

### 5B: Disseminate Knowledge

| **Knowledge Dissemination**  It is outside the scope of this guide to offer specific direction around disseminating knowledge, and there are many high-quality sources on responsible [23], effective [24], and innovative [25] knowledge dissemination. | **How will you prepare to disseminate knowledge?** |
| --- | --- |

References

[1] T. C. Hoffmann *et al.*, “Better reporting of interventions: template for intervention description and replication (TIDieR) checklist and guide,” *BMJ*, vol. 348, no. mar07 3, pp. g1687–g1687, Mar. 2014, doi: 10.1136/bmj.g1687.

[2] M. Campbell, S. V. Katikireddi, T. Hoffmann, R. Armstrong, E. Waters, and P. Craig, “TIDieR-PHP: a reporting guideline for population health and policy interventions,” *BMJ*, p. k1079, May 2018, doi: 10.1136/bmj.k1079.

[3] P. Nilsen and S. Birken, *Handbook on Implementation Science*. Edward Elgar Publishing, 2020. doi: 10.4337/9781788975995.

[4] B. Field, A. Booth, I. Ilott, and K. Gerrish, “Using the Knowledge to Action Framework in practice: a citation analysis and systematic review,” *Implement. Sci.*, vol. 9, no. 1, p. 172, Dec. 2014, doi: 10.1186/s13012-014-0172-2.

[5] M. Chinman, P. Ebener, P. S. Malone, J. Cannon, E. J. D’Amico, and J. Acosta, “Testing implementation support for evidence-based programs in community settings: a replication cluster-randomized trial of Getting To Outcomes®,” *Implement. Sci.*, vol. 13, no. 1, p. 131, Dec. 2018, doi: 10.1186/s13012-018-0825-7.

[6] S. S. Rogal *et al.*, “Getting to implementation: a protocol for a Hybrid III stepped wedge cluster randomized evaluation of using data-driven implementation strategies to improve cirrhosis care for Veterans,” *Implement. Sci.*, vol. 15, no. 1, p. 92, Dec. 2020, doi: 10.1186/s13012-020-01050-7.

[7] L. A. Palinkas, S. M. Horwitz, C. A. Green, J. P. Wisdom, N. Duan, and K. Hoagwood, “Purposeful Sampling for Qualitative Data Collection and Analysis in Mixed Method Implementation Research,” *Adm. Policy Ment. Health Ment. Health Serv. Res.*, vol. 42, no. 5, pp. 533–544, Sep. 2015, doi: 10.1007/s10488-013-0528-y.

[8] S. Brinkmann, *Qualitative Interviewing*. Oxford University Press, 2013. doi: 10.1093/acprof:osobl/9780199861392.001.0001.

[9] P. A. Adler, P. Adler, and R. S. Weiss, “Learning from Strangers: The Art and Method of Qualitative Interview Studies,” *Contemp. Sociol.*, vol. 24, no. 3, p. 420, May 1995, doi: 10.2307/2076552.

[10] I. Acocella and S. Cataldi, *Using Focus Groups: Theory, Methodology, Practice*. 1 Oliver’s Yard, 55 City Road  London  EC1Y 1SP: SAGE Publications Ltd, 2021. doi: 10.4135/9781529739794.

[11] N. Mays and C. Pope, “Qualitative Research: Observational methods in health care settings,” *BMJ*, vol. 311, no. 6998, pp. 182–184, Jul. 1995, doi: 10.1136/bmj.311.6998.182.

[12] D. Oswald, F. Sherratt, and S. Smith, “Handling the Hawthorne effect: The challenges surrounding a participant observer,” *Rev. Soc. Stud.*, vol. 1, no. 1, pp. 53–74, Nov. 2014, doi: 10.21586/ross0000004.

[13] G. M. Fix, B. Kim, M. A. Ruben, and M. B. McCullough, “Direct observation methods: A practical guide for health researchers,” *PEC Innov.*, vol. 1, p. 100036, Dec. 2022, doi: 10.1016/j.pecinn.2022.100036.

[14] L. A. Palinkas and D. Zatzick, “Rapid Assessment Procedure Informed Clinical Ethnography (RAPICE) in Pragmatic Clinical Trials of Mental Health Services Implementation: Methods and Applied Case Study,” *Adm. Policy Ment. Health Ment. Health Serv. Res.*, vol. 46, no. 2, pp. 255–270, Mar. 2019, doi: 10.1007/s10488-018-0909-3.

[15] E. R. Haines *et al.*, “Ethnography and user-centered design to inform context-driven implementation,” *Transl. Behav. Med.*, vol. 12, no. 1, p. ibab077, Jan. 2022, doi: 10.1093/tbm/ibab077.

[16] E. P. Finley *et al.*, “Periodic reflections: a method of guided discussions for documenting implementation phenomena,” *BMC Med. Res. Methodol.*, vol. 18, no. 1, p. 153, Dec. 2018, doi: 10.1186/s12874-018-0610-y.

[17] A. Grant, *Doing your research project with documents: a step-by-step guide to take you from start to finish*. Bristol: Policy Press, an imprint of Bristol University Press, 2022.

[18] L. M. Rea and R. A. Parker, *Designing and conducting survey research: a comprehensive guide*, Fourth edition. San Francisco, CA: Jossey-Bass, a Wiley brand, 2014.

[19] C. E. Hill, B. J. Thompson, and E. N. Williams, “A Guide to Conducting Consensual Qualitative Research,” *Couns. Psychol.*, vol. 25, no. 4, pp. 517–572, Oct. 1997, doi: 10.1177/0011000097254001.

[20] C. E. Hill, S. Knox, B. J. Thompson, E. N. Williams, S. A. Hess, and N. Ladany, “Consensual qualitative research: An update.,” *J. Couns. Psychol.*, vol. 52, no. 2, pp. 196–205, Apr. 2005, doi: 10.1037/0022-0167.52.2.196.

[21] T. C. Guetterman, M. D. Fetters, and J. W. Creswell, “Integrating Quantitative and Qualitative Results in Health Science Mixed Methods Research Through Joint Displays,” *Ann. Fam. Med.*, vol. 13, no. 6, pp. 554–561, Nov. 2015, doi: 10.1370/afm.1865.

[22] J. D. Smith, D. H. Li, and M. R. Rafferty, “The Implementation Research Logic Model: a method for planning, executing, reporting, and synthesizing implementation projects,” *Implement. Sci.*, vol. 15, no. 1, p. 84, Dec. 2020, doi: 10.1186/s13012-020-01041-8.

[23] R. Ravinetto and J. A. Singh, “Responsible dissemination of health and medical research: some guidance points,” *BMJ Evid.-Based Med.*, vol. 28, no. 3, pp. 144–147, Jun. 2023, doi: 10.1136/bmjebm-2022-111967.

[24] L. E. Ashcraft, D. A. Quinn, and R. C. Brownson, “Strategies for effective dissemination of research to United States policymakers: a systematic review,” *Implement. Sci.*, vol. 15, no. 1, p. 89, Dec. 2020, doi: 10.1186/s13012-020-01046-3.

[25] T. Ross-Hellauer *et al.*, “Ten simple rules for innovative dissemination of research,” *PLOS Comput. Biol.*, vol. 16, no. 4, p. e1007704, Apr. 2020, doi: 10.1371/journal.pcbi.1007704.
